# A Review of Activity Trackers for Senior Citizens: Research Perspectives, Commercial Landscape and the Role of the Insurance Industry

**DOI:** 10.3390/s17061277

**Published:** 2017-06-03

**Authors:** Salvatore Tedesco, John Barton, Brendan O’Flynn

**Affiliations:** Tyndall National Institute, University College Cork/Lee Maltings, Prospect Row, Cork T12R5CP, Ireland; john.barton@tyndall.ie (J.B.); brendan.oflynn@tyndall.ie (B.O.)

**Keywords:** wearable sensors, physical activity, healthcare, insurance, older adults, activity trackers

## Abstract

The objective assessment of physical activity levels through wearable inertial-based motion detectors for the automatic, continuous and long-term monitoring of people in free-living environments is a well-known research area in the literature. However, their application to older adults can present particular constraints. This paper reviews the adoption of wearable devices in senior citizens by describing various researches for monitoring physical activity indicators, such as energy expenditure, posture transitions, activity classification, fall detection and prediction, gait and balance analysis, also by adopting consumer-grade fitness trackers with the associated limitations regarding acceptability. This review also describes and compares existing commercial products encompassing activity trackers tailored for older adults, thus providing a comprehensive outlook of the status of commercially available motion tracking systems. Finally, the impact of wearable devices on life and health insurance companies, with a description of the potential benefits for the industry and the wearables market, was analyzed as an example of the potential emerging market drivers for such technology in the future.

## 1. Introduction

Population ageing, and the related increase in chronic diseases, is having a major impact on the healthcare systems of most western countries, and will be having an even more significant effect in the future. For example, as indicated by the United Nations in 2015 in [1], by 2030 the number of older persons (defined as those aged 60 years or over) in the world is projected to grow by 56%, from 901 million to more than 1.4 billion. Likewise, the global population aged 80 years or over is projected to grow from 125 million in 2015 to 202 million in 2030 and to 434 million in 2050. Even though the ageing process affects virtually all countries, it is particularly significant in Europe and North America where, by 2030, older persons are expected to account for more than 25% of the population. As reported in [1] “*As populations continue to age during the post—2015 era, it is imperative that Governments design innovative policies specifically targeted to the needs of older persons, including those addressing housing, employment, health care, social protection, and other forms of intergenerational support. By anticipating these demographic shifts, countries can enact policies proactively to adapt to an ageing population*”. Together with this ageing process, it is evident that the burden of chronic diseases on health systems internationally is rapidly increasing, as indicated by the World Health Organization [2]. In this report it has been calculated that, in 2001, chronic diseases contributed approximately 60% of the 56.5 million total reported deaths in the world and approximately 46% of the global burden of disease. Almost half of the total chronic disease deaths are attributable to cardiovascular diseases. Obesity and diabetes are also showing worrying trends, not only because they already affect a large proportion of the population, but also because they have started to appear earlier in life. It has been projected in [2] that by 2020 chronic diseases will account for almost three-quarters of all deaths worldwide. The number of people in the developing world with diabetes will increase from 84 million in 1995 to 228 million in 2025. As for the number of overweight and obese people, not only has the current prevalence already reached unprecedented levels, but the rate at which it is annually increasing in most developing regions is substantial.

Chronic diseases could be prevented through healthy diet, avoidance of tobacco products, and regular physical activity [3]. However, when manifested, chronic diseases result in limitation of mobility and physical activity of the affected persons [4], with a slow, progressive, and sometimes unnoticed entry mechanism. Usually, the only observable effects are the reduction of the level of autonomy and the loss of mobility. Thus, as a consequence, monitoring physical activity is a valuable parameter in order to define if persons are performing enough physical activity in order to prevent chronic disease or if they are manifesting early symptoms of those diseases [5].

A reliable and continuous 24 h measurement and recording of the physical activity in daily life is thus essential and several solutions have been proposed, from video to smart homes, whose deployment remain intrusive and expensive. Therefore, the use of micro sensors worn by elderly people to analyse body movements [6] seems an acceptable solution for the individuals and their caregivers, and such a solution has been investigated by researchers in the last years thanks to the massive diffusion of micro electromechanical systems, which made those micro sensors highly available, miniaturized, and low-cost.

The present work is organized as follows: a comprehensive review on the physical activity indicators is provided in Section 2, while the tools and technologies adopted for assessment are described in Section 3. Various challenges and the limitations of those methods when applied to senior citizens are then reviewed in Section 4, with a subsection dedicated to each indicator. Moreover, a specific subsection is dedicated to the accuracy of general commercial products and also their acceptability by the senior citizen population. Finally, this paper reviews and compares existing commercial products in the area to provide an outlook of the current development status (Section 5), considering both marketed and forthcoming systems, along with a discussion on the role of insurance companies into the wearables market with the aim of increasing the usage of those technologies (Section 6).

## 2. Physical Activity Indicators

In this section, several common indicators of activity behaviour are described. Those indicators continuously measure long-term activities in subjects in free-living environments and can be used to identify posture and classify daily activities correlated to the subject’s functional status. Moreover, other useful information can be obtained by the energy expenditure. Gait analysis and balance evaluation are also widely studied [7] in order to provide information on subject’s mobility and evaluate the risk of falling. Fall detection is another important aspect to be considered. An example of the indicators commonly taken into account by clinicians and researchers may include:

Physical activity assessment and sedentary behaviour measurement;
Posture detection with related postural transition times estimation;
Daily activity classification;
Energy expenditure and exercise intensity estimation;
Fatigue detection;
Detection and prediction of falling events;
Gait analysis and related balance/stability evaluation;
Detection of abnormal characteristics (tremor, freezing event, etc.…);
Sleep analysis;
Location-awareness information.

### 2.1. Sedentary/Activity Monitoring and Activity Classification

Sedentary behaviour, defined as “any waking behaviour in a sitting or reclining posture with an energy expenditure ≤1.5 metabolic equivalent (MET)” [8], is a dominant behaviour nowadays which is connected to a high risk of developing chronic disease. Physical activity, instead, is defined as “any body movement produced by the skeletal muscles that results in a substantial increase over resting energy expenditure” [9]. Its benefits in preventing chronic disease are currently well-known and it is generally categorized into light intensity (2 to 3 METs), moderate (3 to 6 METs) and vigorous (>6 METs). Generally, a parameter provided to indicate the amount of physical activity performed during the day is the number of steps taken. Step counting on its own, however, does not measure the intensity, duration or frequency of physical activity, which, additionally, does not allow the evaluation of physical activity patterns within or across days.

However, it has been also reported that the amount of time spent sedentary may be an important health risk factor for all-cause mortality, independent of physical activity levels [10].This conclusion suggests to focus on both the accumulation of moderate-to-vigorous physical activity, the reduction of sitting times, and the importance of sedentary/physical activity distribution throughout the waking hours. It is thus essential to estimate accurately the amount of time spent in a sedentary activity, its classification, and its distribution throughout the day. Typical sedentary activities involve lying, sitting, and standing, which can be distinguished by observing the different characteristics of the body segments. Moreover, postural transitions (and, in particular, sit-stand transitions) can be specific indicators to assess the quality of life of people with mobility problems. On the other side, general daily activities of interest may encompass walking, running, ascending and descending flights of stairs, etc.… However, greater attention is currently focussed on fine-grained classification by considering more granular daily movements, such as reading, socializing, watching TV, or playing video games (as per sedentary behaviour), and brushing teeth, dressing/undressing, taking medication, preparing breakfast, house-cleaning, plant watering, or clothes folding for what concerns daily activities.

### 2.2. Energy Expenditure

Energy expenditure is expended as a result of physical activity and is extremely important in the context of the increasing rates of obesity, type 2 diabetes mellitus, and other diseases. This indicator can then be estimated by measuring physical activities and can be expressed relative to resting values where 1 MET at rest equates to 3.5 mL/O_2_/kg/min^−1^ or 1 kcal/kg/h. Gross energy expenditure is quantified on the basis of oxygen consumption and is referenced in kilocalories per minute, kilojoules per minute, or indices that are expressed relative to body mass [11]. Energy expenditure is usually indicated indirectly through inference from activity counts per defined period of time (epochs). Activity counts represent the estimated intensity of measured activities during each time period and can be translated into estimates of energy expenditure using regression-based models [12]. Intense physical exercise might lead to fatigue, defined as the temporary physical inability of a muscle to perform optimally. This condition occurs gradually, and depends upon an individual’s level of physical fitness, and other factors, such as sleep deprivation and overall health, and has been known to be one of the factors impacting on the risk of falling events, especially in older adults [13].

### 2.3. Fall Detection and Gait/Balance Analysis

Fall-related injuries can rapidly deteriorate the health and functional status of elderly people, leading to loss of independence and higher risk of morbidity and mortality [14], and for older people represent a serious health problem, with more than 30% of people older than 65 suffering a fall at least once a year [13]. Detection of falling events have the potential to mitigate the adverse consequences of a fall and can have a direct impact on the reduction in the fear of falling and the rapid provision of assistance, thus reducing the time the subject remain lying after falling. This time is one of the key factors that determine the severity of a fall and is a marker of weakness, illness and social isolation and is associated with high mortality rates among the elderly [15]. Given the unique patterns and characteristics that define falling events, wide attention is also currently being paid in predicting these events and specifying the probability that a subject may suffer a fall in a particular period of time in the short future.

Falls can be also caused by other risk factors, such as fear of falling, medication, vision and sensory problems, ageing, postural stability, and commonly balance control and gait stability. In a balance test, postural sway (e.g., the horizontal movement of the centre of gravity and the related trajectory) is one of the main indicators of the potential risk of falling. Gait disorders are also among the most common causes of falls in older adults and can be an early sign of chronic disease. Gait and balance disorders are usually multifactorial in origin and the early identification and an appropriate intervention may prevent dysfunction and loss of independence. It has been reported that, in a sample of noninstitutionalized older adults, 35% were found to have an abnormal gait. This value increases with age and is higher in persons in an acute hospital setting and in those living in long-term care facilities [16]. Significant gait parameters have been shown to provide an indication regarding the difference between young and elderly subjects; such as a reduction in gait velocity and stride length in the older population, an increased stance width, increased time spent in the double support phase, higher variability, reduced movement smoothness and symmetry, and less vigorous force development at the moment of push off [16]. The appearance of those senile gait patterns may actually represent an early manifestation of subclinical disease.

Even though determining that a gait is abnormal can be challenging, as there are no clearly accepted normal gait standards defined for older adults, it is important for clinicians to detect specific features or abnormalities which may mark the onset of particular conditions. For instance, tremor in the upper limbs, freezing events in subjects with Parkinson’s disease, wandering in people with Alzheimer’s disease, and so on, are all useful indications of health which can be detected and studied.

### 2.4. Sleep Analysis

Sleep disturbances are also very common in older people. Those sleep disorders decrease the quality of life and can be linked with premature death. Even though sleep structure and duration can be significantly altered throughout the aging process [17], there are several factors which can influence those disorders, such as physical illness or symptoms, medications, changes in activity or social life, etc.… Several indicators can be used to describe those disturbances, including: sleep latency, the number and duration of nocturnal awakenings, the total sleep time, changes in the number and rhythms of particular sleep stages (i.e., REM and non-REM states), recurring nights of sleep disruption, and detection of respiratory and snoring features for apnoea diagnosis [18]. Given that sleep analysis is a very extensive research topic that should be addressed more thoroughly, a number of reviews in the area has been included as a reference for the interested readers [19,20,21,22].

### 2.5. Location-Awareness Information

Finally, it has been reported that the availability of contextual information (e.g., location, purpose of behaviour) can be incorporated with the other indicators to provide researchers with the ability to assess the indoor and outdoor location of physical activity and sedentary behaviour [23]. For instance, GPS, wearable cameras, and wireless communication technologies (UWB, RFID, Wi-Fi, etc.…) can be used for this purpose.

## 3. Physical Activity Monitoring and Assessment

Several suitable methods of assessing physical activity and sedentary behaviour have been adopted in the last number of years. In order to define which of those methods should be implemented for a specific case the following factors should be considered [24]:

1. Purpose of the assessment, such as epidemiological research, specific populations physical activity monitoring, physical activity correlates and determinants definition, health programs effectiveness measurements, and so on;
2. Target population, e.g., pre-schoolers, children, teenagers, elderly, people with chronic diseases, or general adult population;
3. Components of physical activity being measured, which include the frequency, intensity, amount, type and setting of activity;
4. Practicality of the measurement tool, referring to the development, administration, scoring, and administration of an assessment;
5. Participant burden;
6. Reliability and validity of the tool being used, indicating the stability of the tool to measure the same concept over time and how well the tool assesses what it is intended to assess, respectively.

In adults, a typical method of collecting data on physical activity is by self-administrated questionnaires, or by some form of direct measurement of movement.

### 3.1. Self-Report

A simple tool for physical activity assessment is by self-report, through the completion of questionnaires, interviews and surveys, or, alternatively, physical activity diaries or logs are used where information on all forms of activity is recorded each day. Those tools require a detailed description of the activity performed, its’ intensity and duration which might be implemented via a 24 h recall plus an extrapolation to the previous days to arrive at an impression about the habitual activity levels of the subject. A diary based method of recording what an individual actually does on a daily basis could produce useful health related data, but this approach requires considerable dedication by the observer. The recording of relevant data has to be relatively simple and extended to cover several days in order to avoid any potential bias to the results. Some well-known examples of those tools are: the Godin Leisure Time Questionnaire (GLTEQ), the International Physical Activity Questionnaire (IPAQ) in its short and long form, the Sedentary Behaviour Questionnaire (SBQ), Baecke’s Physical Activity Questionnaire, Follick’s Diary, the Minnesota Leisure Time Physical Activity Questionnaire, the Physical Activity Scale for the Elderly (PASE), the Zutphen Physical Activity Questionnaires (ZPAQ), the Stanford Usual Activity Questionnaire, the Stanford Brief Activity Scale, the Stanford 7-day Physical Activity Recall (PAR) scale, the Paffenbarger Physical Activity Questionnaire, the Saltin and Grimby Questionnaire, the Yale Physical Activity Survey, and the Quantification de l’Activité Physique (QUANTAP). Those tools are all listed in Reference [25]. However, it is evident that those instruments can provide only qualitative outcomes. In order to overcome those disadvantages and achieve direct, objective and reliable information on physical activity, several alternative techniques have been studied.

### 3.2. Video-Recording

Video-recording, adopting static cameras, wearable cameras or low-cost systems (such as the Microsoft Kinect) is an example of an autonomous data collection methodology [26,27]. Even though it has a definite role in the assessment of activity patterns with the advantage of direct observation, this technique is unlikely to be practicable for large groups of individuals requiring a great amount of resources to analyse and quantify the video-recordings.

### 3.3. Smart Home and Ambient Assisted Living (SHAAL)

Smart Home and Ambient Assisted Living (SHAAL) systems utilize advanced and ubiquitous technologies including sensors and other devices (infrared, pressure mats, automatic bedroom lights, biosensors for vital signs, temperature monitors, etc.…) integrated in to the residential infrastructure, to capture data describing activities of daily living and health-related events [28]. SHAAL systems can increase access to information for older adults and their families, and capture information about activities of daily living, well-being and social interactions in actual residential settings. These data sets can expand the knowledge base of researchers beyond clinical or strictly physiological variables assessed within clinical settings, thus providing more meaningful and representative knowledge basis for shared decision-making. However, it is evident that those systems are expensive and can be solely adopted for a small cohort of subjects.

### 3.4. Doubly Labeled Water (DLW), Indirect Calorimetry, and Heart-Rate Recording

The classic gold-standard techniques for measuring energy expenditure are based on the doubly labeled water method (DLW) or indirect calorimetry measuring oxygen uptake, carbon dioxide production and cardiopulmonary parameters. Though accurate, indirect calorimetry is expensive and requires specialized training, and likewise the DLW method is costly and not suitable for large-scale studies [14]. On the other side, heart-rate monitors are low-cost and can provide information on heart-rate. However, while heart-rate can be a good general indicator of activity, it is not a precise indicator of energy expenditure, unless a proper individual calibration is performed [29]. Moreover, analysing heart-rate records often requires some expert decision-making and is a tedious procedure.

### 3.5. Polysomnography (PSG)

Polysomnography (PSG) is the criterion for objective sleep monitoring and is usually performed in specialized centres or hospitals [30]. PSG typically records multiple electrophysiological parameters, such as EEG, EOG, EMG, and ECG, which are analysed by experts. Even though PSG provides an accurate assessment of the sleep structure, its high cost makes the PSG impractical to be used as a long-term monitor or in large-studies. In addition, the use of several electrical wires attached to the body may, in turn, disturb sleep, thus affecting the measured sleep behaviour of subjects.

### 3.6. Motion Detectors

In order to avoid the issues typical of gold-standard technology and guarantee low-cost, accurate and reliable data, wearable motion detectors have been commonly used in clinical and research settings for objective physical activity measurement. Pedometers are step counter devices designed to measure vertical movement. They have been in use for many years, but pedometers are not accurate when used for activities that do not involve footfalls (such as swimming or upper body movements). Moreover, accuracy may be poor among specific populations, and among different models [31]. Accelerometers are technically more advanced than pedometers and being multi-axial can measure horizontal, lateral, and vertical movements. The reliability and validity of accelerometer data is generally high, and it has been proven that those devices can be used to measure steps, activity counts, energy expenditure, posture, walking, and different intensities of movement. Moreover, accelerometers can be worn by the subjects for days at a time without issues. However, interpreting data from accelerometers can be challenging and varies according to age, gender, functional levels, and health status of the subjects [12]. Indeed, the cut-points for intensity of activities vary according to age, gender, functional levels and the type of accelerometer being worn. Additionally, performance of the motion sensor can vary according to the location chosen for the device placement, such as trunk, lower back, hip, waist, thigh, shank, or foot [32,33].

Recently, significant attention has been paid to wrist-worn accelerometers as they are convenient and comfortable to wear, as well as improve compliance in studies where there is prolonged wear time. At the same time these systems provide a high level of accuracy.

Furthermore, the integration of additional motion sensors is taken into account to increase the overall performance. Such integrated sensors may include gyroscopes, and magnetometers, but also barometers, GPS and physiological sensors (e.g., heart rate) devised to improve the assessment/detection of specific indicators [34,35,36]. The interested readers may also refer to Reference [37,38,39] for an in-depth analysis of the working principles of the mentioned sensors.

A number of reviews studying the adoption of wearable motion sensors for physical activity monitoring and assessment are currently available in literature [12,14,40,41,42,43,44], however, little to no consideration is given to wearables for older adults. A selected literature review, which satisfies this requirement, will be described in Section 4.

## 4. Wearables for Senior Citizens: Related Works and Limitations

Researchers’ interest has covered several aspects of the usage of motion detectors in the older population. The main research topics can be differentiated into:

- Realization of prototypical activity trackers and the implementation of remote monitoring infrastructures;
- Definition of advanced algorithms for physical activity monitoring, posture transitions analysis, fall detection, gait and balance analysis, etc.… with the related validation on geriatric samples;
- Investigation on smartphones-based monitoring systems;
- Adoption of consumers’ fitness trackers on older subjects, accuracy of those systems, and study of the attitude towards those devices.

Those areas will be described in detail in the following subparagraphs.

### 4.1. Remote Monitoring Systems

Earlier published works studied the realization of specific “ambulatory activity recorders” for older adult subjects to be worn for physical monitoring. Examples include the works published by Noury et al. [45,46] for the ACTIDOM project, combining 3D accelerometers and 3D magnetometers, and the CAALYX project, which aimed to develop an electronic device (or “smart garment”) able to detect falls of older subjects in the domestic environment and outside [47].

Researchers also investigated the development of a body wireless network infrastructure requested to connect motion detectors, and potentially additional sensing technologies, to a base station or beacon (such as a PC, or a smartphone) which, after processing the data, can consequently send alerts to caregivers, assessing a tele-medicine approach. Initial examples in the previous decade of similar infrastructures were:

- Reference [48], where an Intelligent Accelerometer Unit (IAU), fixed to the patients back, at the height of the sacrum, transmits signals to a WPAN server (PSE) for online processing, supported by a wireless personal area network WAN, for full 24 h supervision;
- Reference [49], where a dual-axis accelerometer measures body movements produced by respiration, posture changing, falling, and activities, and if the person’s respiration is paused for 3 min, or if they are in an inactive state for 1 min after falling, or for 64 min without previously falling, then the system automatically sends the person’s location to the family by e-mail or phone;
- Reference [50], where a wearable device including accelerometer, gyroscope, and heart rate, worn on the patient’s chest transmits signals via ZigBee to a wireless receiver connected to a laptop, on which a fall detector algorithm is implemented. The software on the laptop is able to send alarms to caregivers/relatives via Internet in case of necessity.

Integration of multiple sensors into the body sensor network for vital sign monitoring were also considered in [51,52,53,54], including sensors for respiratory rate, skin temperature, surface EMG, ECG, and plantar pressure. However, despite the advantage given by the multitude of sensors providing an overall detailed picture related to the physical status of the subjects, such systems may be considered obtrusive for elderly people. For this reason, [55] described a solution where activity, respiration rate, heart rate, posture and behaviour were estimated from only one accelerometer worn on the chest, which then transmits outcomes to a server remotely available by the caregivers. Likewise, [56] investigated the adoption of a watch-like device, including an accelerometer, which detects the physical activity and stores data locally and periodically tries to connect to the data gateway which, in turn, try to send the measurements to a remote database where data is stored and analysed by end-users. In this study, the compliance and acceptance of the system was greater than that which was described in other works. Similar conclusions were drawn in [57] with a waist-like device used by older people with chronic kidney disease. However, in both studies, the population samples were limited as they involved only three and five subjects, respectively.

Other examples of network infrastructures or frameworks for behaviour analysis and fall detection in the elderly are shown in [58,59,60,61]. A recent work [62], extended the system architecture by including mobile cloud computing implemented on an Android device for sending alerts, yet adopting an easy-to-use and familiar user-interface.

Finally, a real-world application of those remote monitoring systems has been published in [63]. In this case, a necklace tag with an accelerometer is able to process data and discriminate among five main postures. Results are transmitted to a base station, and then to the caregivers which receive alarms on a wireless enabled watch. The system has been tested on 20 patients in a nursing home. A comparison between the listed works is shown in Table 1.

### 4.2. Physical Activity Assessment and Energy Expenditure

There are several issues concerning physical activity measurement that are unique to the older adult population. Firstly, older adults present differences in the type and intensity of activities compared to younger adults and children [64]. Moreover, chronic conditions, typical of old age, can affect physical activity levels. This section of the document seeks to provide information on studies conducted using an accelerometer in older adult samples, despite the lack of a standard evaluation protocol among those investigations [41,65]. Indeed, several aspects should be considered when designing this type of assessment: the type of accelerometer used, its placement, the number of days the device was worn, compliance of the subjects, physical characteristics of the population (such as impaired or unimpaired subjects, economical status, geographical provenience, etc.…), and outcomes (e.g., activity counts, energy expenditure,…).

Even though custom algorithms can be considered to estimate physical activity in elderly [56], research-grade accelerometers are usually deployed in clinical studies, which however do not provide details on the employed algorithm in the particular systems described. Examples of those devices are the ActiGraph [66], ActiWatch [67], ActivPAL [68], DynaPort [69], and many others. Those studies have been used in a wide number of investigations, such as

- Definition of the pattern of physical activity and sedentary behavior in older people and the quantification of moderate-to-vigorous activity, [70,71];
- Evaluation of physical activity in older samples with particular conditions (schizophrenia [72], multiple sclerosis [73], frailty [74], hospitalized subjects [75,76], severe aortic stenosis [77], acute and subacute stroke [78]);
- Correlation of physical activity with subclinical vascular disease [79], disabilities [80,81], cognitive function [82,83], cardio-metabolic risk [84], bone density [85], or lean mass percentage [86];
- Quantification of the effects that specific life events may have on physical activity, such as stressful events [87], or retirement [88];
- Geographical differences of physical activity, investigated in Norway [89], the UK [90,91], Iceland [92], China [93], Japan [94], and dissimilarities between subjects in urban and rural contexts [95,96].

However, a limitation of those research-grade devices is given by the definition of the cut-points corresponding to the different METs used for discriminating between sedentary, light, and moderate-to-vigorous activities, which may vary according to the population age. It has been reported in Reference [97] that selected assumptions on the cut-points used in analyzing accelerometer counts can produce markedly different results, and using too low or too a high cut-point may obscure important group or treatment differences. To this purpose, [98] derived specific cut-points for older people while walking, suggesting that older adults are likely to have higher activity levels than reported by previous observational studies using younger adult defined cut-points.

It is well-known that activity counts can also be correlated with subjects’ energy expenditure and oxygen uptake. Energy expenditure in a young healthy population has been studied extensively, however, applying such estimation models to older subject groups needs to be investigated. Examples on this area consider the adoption of a wrist-based device [99], a waist-attached sensor combined with a heart rate monitor validated on older adults with multiple sclerosis [100], or lower-limbs accelerometer measures [101], achieving accurate predictions.

### 4.3. Activity Recognition and Posture Transitions

#### 4.3.1. Activity Classification

Another important aspect when assessing functional status in older adults concerns the discrimination of the activities performed, which can provide additional information on the elderly monitoring. In earlier works, basic body postures (sitting, standing, lying), standard activities (walking, running), and the related posture transitions (e.g., sit-to-stand, stand-to, sit, etc.…) were usually considered. For example, Najafi et al. [102] developed a method based on wavelet transform, in conjunction with a simple kinematics model, to detect postures and transitions using only one kinematic sensor (consisting of a 1D gyro, and two 2D accelerometers) attached to the chest, while Reference [103] adopted two 2D accelerometers attached to thigh and sternum to achieve an accuracy in detecting static postures of 92% via an inclination-based thresholding algorithm. Other approaches included a Dynamic Time Warping (DTW) method applied in [104] using a 3D accelerometer attached on the user’s waist and showing 91% accuracy in recognizing the movements, while a binary tree algorithm was shown in Reference [105] with a similar set-up. An interesting algorithm was described in Reference [106] where a combination of different neural networks, and linear discriminant analysis were used to recognize activities in senior citizens with an average accuracy of 94% and without requesting a firm attachment of the sensing device on the body, which could be regardless worn on the front/rear trouser pockets, chest pockets and the inner jacket.

Moreover, the classification of different walking patterns, including ascending and descending stairs in an unconstrained scenario, suitable for real-time applications, has been considered. As an example, [107] achieved this by applying a wavelet transform to the acceleration signal recorded at the waist, while Muscillo et al. [108] adopted a naïve 2D-Bayes classifier with a correct classification rate higher than 92%. It has been proven in [109] that a single sensor with an accelerometer and gyroscope attached to the lower back is able to perform the classification.

Another important aspect in activity recognition is related to the classification of activities of daily living (ADL), to be integrated with the ambulatory monitoring described above. ADLs cover a wide range of activities typically carried out in daily living, and are usually related to activities such as feeding, watching TV, using a telephone, cooking, etc. For instance, in [110] several features were extracted from a multitude of sensors (a heart rate monitor worn over the chest, accelerometer, gyroscope, light, and barometer sensors attached to the dominant wrist, and temperature sensor and altimeter worn on the other wrist) and given as input to three machine learning methods whose outcomes were merged by adopting a genetic algorithm defining the fusion weights. The activities taken into account included brushing teeth, exercising, feeding, ironing, reading, scrubbing, sleeping, using stairs, sweeping, walking, washing dishes, watching TV, and wiping, and the best combination of classifiers achieved an accuracy of 98%. Another work [111] adopted a wide range of physiological sensors (e.g., electrodermal activity and photoplethysmogram) combined with an accelerometer on the wrist, in a machine learning algorithm to assess cognitive impairment in older people with dementia. Outcomes were statistically matched with clinical scores in order to improve automatic recognition. However, the required number of sensors can be obtrusive for the subjects, therefore adopting only a wrist-based accelerometer (considered more socially-acceptable) has been further investigated. Sasaki et al. [112] used accelerometer data collected from a wrist-sensor to train random forest and support vector machine algorithms in order to discriminate household, locomotor, and recreational activities. Even though results were not sufficiently accurate, a significant difference in system performance was observed when the algorithms were trained with free-living data rather than laboratory data. On the other hand, [113] has proven the feasibility to discriminate between walking and other daily activities with a wrist-based accelerometer. A schematic comparison between the mentioned papers is shown in Table 2.

#### 4.3.2. Posture Transitions Estimation

While the continuous observation of activity patterns throughout the whole day is important, analysing the transitions between those activities is also essential so as to provide useful information on the functional status of a subject. Indeed, rising from a chair can be one of the most demanding activities for elderly people. Several studies analysed the dynamic and kinematic variables in the postural transitions. For example, [114] adopted a fractal dimension of the acceleration-angular velocity plot with data collected from a trunk movement to quantify the effects of rehabilitative care on elderly performing sit-to-stand and stand-to-sit transitions. A combination of filters and peak-detection method were instead adopted in [115]. Zhang et al. [116] proved that a pendant-worn sensor could be reliably used for assessing chair rise monitoring. Finally, other published works investigated the possibility to automate the assessment with sensors attached to the lower-back during sit-to-stand [117], sit-to-walk [118], and 30 s chair test [119].

### 4.4. Gait and Balance Evaluation, Fall Detection and Risk Prediction

#### 4.4.1. Gait Analysis

Gait analysis is an essential tool for the assessment of a variety of movement disorders supporting clinical diagnostics and medical treatments, and can thus be especially important to assess the overall quality of life in older adults. For this reason, several algorithms have been studied to define an accurate method to assess gait in older adults. For example, Mariani et al. [120] obtained accurate results with a double integration method of the 3D accelerometer data collected on the shoes of the subjects, followed by a specific de-drifting approach. Similar results were achieved in [121] adopting a different de-drifting model. Normative values of the gait parameters, with particular focus on the foot clearance parameters, were also obtained by more than 1400 able-bodied adults over the age of 65 using those shoe-worn inertial sensors [122]. However, other sensor positionings were proposed which also achieved good results, such as ankle [123], ear [124], and lower-back [125,126]. Those latter works did consider gait when ascending and descending stairs, also implementing a method to discriminate between young and elderly subjects.

Gait-related characteristics were also obtained for older adults population with specific health conditions, such as bilateral knee osteoarthritis [127] and Parkinson’s disease [128], showing differences in gait compared to healthy subjects.

Moreover, a number of validity and reliability studies have been performed adopting commercial systems to senior citizens. For instance, Dynaport [69] has been tested in [129,130] proving that averaged step data are in excellent agreement with criterion, even though gait variability measures and individual steps should be viewed with caution. However, [131] reported that gait variability assessment could be obtained reliably from the inertial data collected on the lower trunk. Moreover, as shown in [132], RehaGait [133] could be used as a valid and reliable tool for assessing spatio-temporal gait parameters for treadmill walking at different speeds and slopes, even though stride length at slow speeds presented a lower reliability. However, most of those tests have been performed by older subjects in laboratory conditions, which, as it has been noticed in [134] adopting a wearable pendant device, may present different gait characteristics in daily life, thus suggesting that novel protocols for testing in free-living environments should be implemented. A schematic comparison between the mentioned papers is shown in Table 3.

#### 4.4.2. Balance Analysis

Balance is another important aspect which affects quality of life, and it is especially important in older people for assessing their functional independence. Validated clinical tools for balance assessment include the Berg Balance Scale (BBS) and the Timed Up and Go test (TUG). However, while the former tends to be subjective and dependent on the experience of the evaluator, the latter quantifies the functional impairment but requiring further subjective evaluation. It has been determined that accelerometry in older people shows a high correlation with those clinical tools [135]. Moreover, [136] defined a set of accelerometer-based parameters for measuring the postural sway and discriminate between young and elderly subjects. The TUG test assessed with a wrist-based accelerometer has been also linked to the prediction of disability levels in community-dwellers older adults [137], while the inertial-based assessment reliability of the TUG via testing measures of variability over 5 continuous days during single-task and dual-tasks was analysed in Reference [138]. Sheehan et al. [139] adopted the same approach with an accelerometer attached to each shank to investigate the monitoring of functioning decline by testing older subjects one year apart. Finally, the possibility to adopt hip and ankle-based sensors to distinguish five different dynamic balance exercises and evaluate their performance via machine learning techniques has been considered in [140].

#### 4.4.3. Fall Detection and Prediction

Gait and balance instability are among the most diffused causes of falls. Falling events may have catastrophic consequences, especially in older people. Indeed, falls are the leading cause of disability, injury-related deaths, and emergency hospital admissions for adults over the age of 65. An extensive amount of studies have been published in the literature [141,142] involving several methods, such as thresholding on the accelerometer signal, trunk inclination evaluation, machine learning, velocity profile evaluation, and so on, using sensors on different locations. However, most of those methods have been tested on simulated falls performed by young subjects. Even though simulated falls have been useful for developing analytical models, it has been shown that the algorithms realized with those events may lack the necessary accuracy requirements for real-world fall detection as they may not mimic real-world falls sufficiently [143,144]. Despite several works deploying wearable sensors on older adults with the aim of capturing real-world falls [145,146], only a few studies [143,144,147,148] managed to record them. In particular, Palmerini et al. [147] developed a wavelet-based approach based on a dataset of real-world falls collected during the algorithm development phase with a sensitivity of 90%, while [148], as part of the FARSEEING project aiming to record the largest database of real-world falls to date using inertial sensors, analysed 100 real-world falls and employed a machine learning approach to discriminate them from usual daily living activity with a sensitivity of 88%. The sensors were attached to the lower back to enable the development of fall detection algorithms tailored to older adults.

Furthermore, significant attention has been paid to the investigation of falls prediction and fall risk evaluation. Despite extensive research, falls are still difficult to predict because of the multiplicity of risk factors involved. Several parameters have been considered for distinguishing fallers and non-fallers behaviour in elderly people. Some of them involve:

Gait rhythmicity [149];
Spectral analysis on accelerometry data during specific exercises [150];
Temporal gait parameters [151];
Gait stability and symmetry [152];
Time and mediolateral acceleration and spectral analysis in a sit-to-stand test [153];
Sway range, sway length and sway velocity while standing [154];
Angle, velocity and acceleration of pelvis movement during walking [155];
Stride dynamics and gait variability [156];
Harmonic ratio, index of harmonicity, multiscale entropy and recurrence quantification analysis of acceleration during gait [157];
Lateral harmonic stability on gait [158];
Wavelet-transform during sit-to-stand [159];
Refined composite multiscale entropy and refined multiscale permutation entropy during walking [160];
Combinations of the previous variables [161,162,163].

However, the populations taken into account in those studies may be biased towards relatively healthy older people. Therefore, additional analyses are required to further establish the predictive ability of those methods also for specific populations.

### 4.5. Smartphone-Based Monitoring Systems

The opportunity to reduce the infrastructure cost of monitoring the elderly by adopting inertial sensors already embedded into nowadays massively diffused smartphones has been investigated previously in relevant literature. Most of the aspects described in Section 4.2, Section 4.3, and Section 4.4 have been reproduced on a mobile phone and validated on older people. For example, physical activity monitoring has been considered in [164,165], while activity recognition has been investigated in [166,167,168]. Quantification of sit-to-stand transitions have been instead studied in [169,170] and gait and balance test analysed in [165,171]. Finally, it has been considered in [172] the possibility to use such a tool to encourage and support older people in staying active by promoting awareness about their physical activity and enhancing motivation.

### 4.6. Consumer Fitness Trackers and Acceptability

#### 4.6.1. Literature Review

There are a number of studies investigating the impact of commercial fitness trackers in older adults. For example, it was reported that trackers were able to positively impact health by increasing physical activity and motivating older obese adults [173] and helping them reduce blood pressure and lose weight [174,175].

An extensively investigated aspect, recently studied, considers older subjects’ attitude and acceptability towards activity trackers and daily activity. As current wearable devices are mainly designed to attract a young, sporty and technical affine group of people, their usability with older users has been questioned.

In particular, in [176], specifications and requirements (in terms of usefulness, attractiveness, usability, comfort and acceptance) for a wrist activity tracker customized for elderly people have been established, as part of the EMERGE project, while several other studies investigated the acceptance of consumer trackers. In some of these papers [177,178], wearables have been substantially accepted by older users showing a motivating effect for engagement in self-monitoring, but a variety of drawbacks were highlighted, such as concerns on the handling of the data provided by the devices and the consequences this feedback has for future behaviour. In addition, the lack of support, and the fact that many of the fitness tracker’s functions were not used while other additional functions were requested, lead to the conclusion that fitness trackers in their current form are not suited for older adults.

Similarly, Preuss et al. in [179,180] acknowledged the positive attitude of older adults towards the adoption of new technologies, however a series of barriers related to human factors and usability were described. These barriers include Consistency and Standards, Visibility of System Status, and Error Prevention and perceived sacrifices because they pose potential costs (financial, time, effort) to users. Several acceptance-promoting strategies have been also suggested with the aim of explaining benefits and minimizing cost, including communicating personal benefits to the older adult population specifically, creating tutorial videos, adding navigation and accuracy hints to initial start-up guides, and allowing for trial-use periods.

Other research papers have shown a significant variability in the users’ acceptance of results. Trackers were seen as beneficial for helping older participants in keeping an active lifestyle in community-dwellers subjects or people with chronic disease [181,182,183], while [184] showed that the acceptability could decrease in the long-term and being limited in subjects older than 80 years. This is also confirmed in [185] where older people with an average age between 86 and 90 years rejected the wearable technology in favour of classical health providers/caregivers, while [186,187] described a series of challenges experienced by senior citizens when using fitness trackers, such as not perceiving the utility of the device, inaccurate results especially regarding step-counting and sleep analysis, the set-up of the device and data interpretation, discomfort, requiring the use of PC or smartphones to sync the data to the website, high mental efforts to install the app and to put the activity tracker into operation, yet recognizing the potential of the technology. To avoid such issues and make user-experience more engaging for older people a novel gamification-based approach has been successfully adopted in [188].

The comparison in acceptance of fitness trackers in rural and suburban older subjects has been instead studied in [189,190]. In both cases wearables were accepted, however, only in suburban subjects the adoption of a fitness tracker on its own involved an improvement of the daily exercise, while this was not evident in rural older people suggesting a need to include a human component in order to obtain a behavioural change in this high-risk population. Moreover, the study showed a preference by seniors for activity trackers worn on the wrist compared to other locations, as well as a high level of inaccuracy in the results when comparing different trackers with each other.

The accuracy level of fitness trackers in elderly people has been tested in a number of papers. For example, [191] shows that a Fitbit^®^ attached to the hip accurately tracks step-counting among healthy community-dwelling older adults, and it was reported in [192] that the Fitbit^®^ may be more accurate for step-counting at slow walking speed when attached on the ankle rather than on the waist. A similar comparison among three trackers has been carried out in [193] taking into account a waist and wrist positioning, resulting in high accuracy at slow speed with a sensor attached at the waist. Moreover, [194] suggested that the Fitbit^®^ may be considered a viable instrument for measuring daily caloric expenditure among older adults, while [195] showed that the StepWatch™, Omron HJ-112, Fitbit^®^, and Jawbone appeared accurate at measuring steps in older adults with non-impaired and impaired ambulation during a self-paced walking test. However, it has been suggested in [196] that the ActivPAL™ attached to the thigh may underestimate in older people with impaired function, especially at slow walking speed, despite an accurate activity detection, while a significantly high error (>60%) can be observed when testing older people with reduced mobility with a Fitbit^®^ Ultra and a Samsung GT-I9300 mobile phone with a pedometer application installed both attached to hip and wrist [197]. Gait disorders, slow walking, or the usage of rollators increased error in step estimation, suggesting that the tested trackers are better suited for adults and healthy older subjects.

#### 4.6.2. Requirements for New Products

In conclusion, wearable devices can certainly help older individuals to lead healthier and safer lives, but designers must take into consideration the differences in ability among such users and the population in general. Similar outcomes have been diffused by the American Association of Retired Persons (AARP) in the Project Catalyst’s report [198]. Project Catalyst is a collaborative effort between Georgia Tech, the AARP, MedStar Health, Pfizer, and UnitedHealthcare, whose initiative is to identify ways to improve wearables devices for 50-plus consumers. Study participants found the trackers beneficial, especially with regards to learning daily activity and sleep patterns, receiving motivation by seeing progress made toward a goal, having their current activity levels confirmed, and finding the device to be easy to use. However, the trackers were also perceived as inaccurate, lacking of instructions, presenting malfunctions, lost data and difficult sync process, and difficult to wear. Based on this research, it is envisaged that the ideal tracker for the consumer aged 50-plus would be:

- Informative, instilling confidence with materials that explain how activity and sleep trackers work and how they support health and wellness goals;
- Simple, with a straightforward set-up process that includes better indicators for opening the package and removing the device as well as more detailed, step-by-step instructions for syncing;
- Accessible, with packaging and support materials that are easy to open and product instructions that are clear and easy to find. Features should accommodate the functional limitations associated with aging, such as lower visual acuity, lower contrast sensitivity and lower capacity for sequence-based memorization activities;
- Invisible, unobtrusively monitoring activity and sleep without discomfort or annoyance and with little intervention needed on the part of the user;
- Instantaneous, giving users a view of progress that is up to date;
- Targeted to consumers aged 50-plus and their activities, with information tailored to their health and wellness goals of achieving positive health and avoiding ill health;
- Meaningfully engaging, with timely notifications of progress.

To this purpose, the most specific recommendations provided by the participants include:

- Be able to detect more biometric data (such as blood sugar, heart rate and caloric intake);
- Feature a more comfortable band;
- Explain how tracking works, so users could feel confident in the accuracy of the data;
- Include a display;
- Be accompanied by better, more detailed instructions;
- Have a nicer-looking design;
- Leverage data monitoring to provide more alerts, such as progress toward goals and identification of a health situation;
- Display time like a watch;
- Be waterproof;
- Report non-health functions;

Therefore, it is essential that wearable device specifically customized for consumers aged 50-plus should be studied so that older people could fully take advantage of the health benefits related to activity and sleep trackers.

It is worth noting that nearly all of the works referred to in Section 4 have been performed as part of some specific clinical research studies (e.g., Parkinson’s disease, frailty, fall detection, etc.…), clinical epidemiological studies, or research into general lifestyle fitness/performance. There can be a large difference in terms of parameters of interest and estimation accuracy, user interface and interpretability of analysis results, monitoring duration/context, between all of these studies and more so between those that are performed using research-grade sensors and the off-the-shelf activity trackers, as in Section 4.6. Powerful, bespoke algorithms can be developed to analyse data when it is easily accessible. This is usually not the case with the commercial products. Section 5, therefore will review wrist-worn devices available for older adults, some extant and some forthcoming to the market, which may be able to merge the two fields by developing commercial products—taking into account the recommendations above—with embedded algorithms that take advantage of multi-modal sensing for personalised activity monitoring.

## 5. Review of Current Products

There are many smartwatches, fitness trackers, and other wearable devices available on the market which are oriented towards active, healthy customers. On the other hand, only a few developers have seen potential in targeting the needs of people who have particular health concerns by proposing products that help older customers maintain an independent lifestyle. This section reviews several commercially available smartwatches/wristbands designed for elderly people to provide a comprehensive outlook of current development status. However, most of those products are provided by start-ups or have been unveiled as prototypes and are still in the development phase, thus are not available for consumers. While Section 5.1 describes smartwatches/wristbands already available on the market, with a specific section for geolocation-focused devices, Section 5.2 is dedicated to forthcoming systems. A list of interesting non wrist-related wearable products for senior citizens is left at the end of the paragraph (Section 5.3) for in-depth analysis.

It is worth noting this review is related to commercial activity trackers which are specifically designed for senior citizens. Therefore, “classical” commercial devices, such as ActiGraph, GeneActiv, MotionWatch, etc., even though used by elderly people in different studies, are not mentioned, as they target researchers studying several areas of activity monitoring and do not consider the ageing population as their main customers.

### 5.1. Marketed Smartwatches

#### 5.1.1. Lively™

Lively™ [199], recently acquired by GreatCall, Inc., combines a safety watch and home sensors to create a monitoring and emergency response system. The watch has a large time display with backlighting and an emergency help button which connects the user to a trained operator, available 24/7, who can send emergency help if needed. Additional features include a customizable watch band, vibrating medication reminders, step counting, waterproof design, easy set-up, and a clip-on accessory for fall detection. The passive home sensors are attached on movable objects around the home (such as pillboxes, refrigerator, doors, etc.…) and measure insightful data on daily behaviour pattern. If abnormalities in the behaviour pattern are observed, an automatic alert is sent to the caregivers. All the information is gathered from the watch and home sensors into a hub deployed in the home using a built-in cellular connection (thus not requiring any Internet connection), and are shown on an online dashboard available on the website or on a smartphone. The battery lifetime is 2–3 months for the watch and 8–12 months for the home sensors. Lively™ is currently available in the US, the UK, Australia, and New Zealand and costs $49.95 for the equipment plus a subscription fee for the service ($34.95 monthly or $359.40 annually). The adoption of a wearable smart unit differentiates the Lively™ system from other elderly-oriented home-based remote monitoring systems, such as GrandCare [200], Healthsense [201], QuietCare [202], or Evermind [203].

#### 5.1.2. Vytality™ by PeakFoqus

PeakFoqus is a company founded in 2014 which produces Vytality™ Apple Watch Edition [204], an app and mobile package solution for the health and safety of seniors and patients. The system is a beta version and costs $499 including a Vytality™ watch—Apple Watch Sport (with different size and bands), an Apple iPhone, two months of Vytality™ Concierge, and the data plan (powered by Verizon, not included in the overall price). The watch contains an automatic fall detection, activity level and heart rate monitoring, location tracking, and an emergency beacon. The Concierge program instead is related to assistance to watch settings and family notifications customization, access to emergency services, watch set-up videos, and additional health, safety, and social features. The system received an honourable mention at the Wearscript Workshop held at MIT Media Lab in March 2014 and has been showcased at Panasonic Lab 1.0 in February 2015.

#### 5.1.3. GPS Locator Watches

There are a number of localisation systems (including wearables and even smartphone apps) which can be used for monitoring the geo-location of people. Some of those devices include Mindme Locate [205], GPS SmartSole [206], iTraq [207], Proximity Button [208], PocketFinder [209], Comfort Zone Check-In Mobile [210], V.ALRT [211], Tweri [212], and Lok8U [213]. They can be attached on different positions on the body, and rely on a combination of GPS and GSM to localize the wearer and remotely transmit the location to the family or caregivers. Those systems can be also used for geo-fencing, in order to signal if the wearer is crossing a pre-defined perimeter. However, typical customers are not only limited to older people, but also include people with cognitive impairments, with dementia or Alzheimer’s disease, subjects with autism, behavioural dysfunctions and learning difficulties, patients in rehab, or recovering from illness or accident, lone workers, children and even pets.

Several companies have started producing localisation systems built-in watches and wristbands, as wrist-based sensors are more socially accepted by older people. Examples of similar products are PAL from project Lifesaver [214], Safe Link [215], Revolutionary Tracker [216], Bluewater Security [217], and Vega bracelet from Everon [218]. However, additional features have been also considered.

Limmex Emergency Watch [219] is manufactured in Switzerland and has an elegant wristwatch. It includes a help button for triggering emergency calls, and a built-in loudspeaker and microphone, as well as localization capabilities. However, a new generation of watches is under development with the consequences of stopping the production of the current version.

Similarly, Soteria from mCareWatch [220] is an all-in-one watch, mobile phone, personal emergency alarm, localization tracker (with GPS, GSM, and Wi-Fi) with geo-fencing, and medical reminder. In case of an emergency Soteria can be linked to a 24 h emergency personal monitoring service with auto-dial up to three different pre-set contact numbers. It also has camera and video capabilities and the possibility to interact with other biometric devices (blood pressure, weight scale, etc.…) via BLE. Finally, it can also link to the ConnectiveCARE platform and mCareWatch smartphone app, enabling both wearers and carers to access all the additional information, support and features the platform offers. However, the system is only available in Australia.

Finally, Clevercare [221] designed a smartwatch able to make and receive phone calls, define medication and task reminders, implement real-time location and geo-fencing, use a help button with 24/7 emergency response, sturdy, lightweight, with 18–24 h battery lifetime, possibility to visualize data on a dashboard from smartphone, tablet or computer via Internet connection, and the setting of inactivity alerts. However, also in this case, this product is available only in New Zealand.

#### 5.1.4. Remarks

It is important to highlight that most of these new devices mentioned in Section 5.1 integrate multi-modal sensing (motion, physiology, context-awareness, etc.…), and some interesting research perspectives may encompass the implementation of studies to investigate the accuracy of monitored data and their reliability especially for senior citizens. Unfortunately, given the relatively young market for activity trackers targeting this population, those investigations are still not available in literature, but it is likely that will be more feasible in the next few years. One important aspect for the future research studies is represented by the access to the raw data, and this is one important requirement often addressed by researchers to product companies, in order to develop advanced analytical methods to maximally exploit the amount of available data.

### 5.2. Forthcoming Watches

CarePredict produces Tempo [222], a wrist-based sensor which comes with interchangeable bands that monitors the wearer’s daily activities and movements through an accelerometer. Data is sent wirelessly to a hub installed in the home and then uploaded to cloud-secured servers where they can be shown to the caregivers on an online dashboard. The device records users’ daily habits, such as walking speed, visits to the bathroom, time spent sitting, standing or lying down, and position within the house, over a seven days period building a baseline. A powerful machine learning algorithm is adopted to track 24/7 even small changes in the patterns of behaviour, which could point toward early signs of health complications, and send proactive alerts. Medication reminders are also included. Nevertheless, customer release has not been announced at the time of publication (2017).

The UnaliWear Kanega Watch [223] is a discreet, stylish, waterproof, voice-controlled device that will provide on-call or automatic emergency assistance, relying on a fall detection algorithm. The device does not use buttons, and does not require a smartphone. It also provides medication reminders and a guard against wandering. This Kickstarter-funded project is currently in product beta development, and it is expected to reach the consumer market late in 2017.

The Allen Band [224] is a slim wristband which can measure heart rate, inactivity level, body temperature, falls, and GPS location. All the data is sent to the monitoring cloud at regular intervals via an Internet connection (Wi-Fi), through a smartphone connected via BLE to the tracker, or via a cellular connectivity option. Each caregiver can ask the cloud monitoring system to alert them based on their own levels of concern, thus eliminating all additional independent monitoring costs. Each Allen Band can send alerts to up to two caregivers at no cost whatsoever by using a help button. The device also has an OK button to eliminate possible false positive falls detection. The system was partially funded with Indiegogo, is currently being developed and tested for sale in the US and available on pre-order.

IN LIFE [225] is a 3-year EU-funded project which aims to prolong and support the independent living of seniors with cognitive impairment, through interoperable, open, personalized and seamless ICT solutions. The smartwatch system developed for the project is in pre-order and assists elderly people in their everyday activities and automatically detects alarming situations (e.g., falls) appropriately sending alerts to caregivers. The smartwatch comes with a pre-installed application, and it is ready to use out of the box. It is water resistant and includes fall detection, help button, normal smartphone features (calls, SMS, Internet, camera), reminders, and localization.

WatchRx [226] is a company founded in 2015 and its all-in-one watch is designed to be able to remind the elderly to take their medications on time, alerting caregivers in case of any missed medications. The watch also provides reminders for daily activities and daily checks-in, alerting caregivers upon no response to these check-ins. The built-in phone allows for emergency calling as well as incoming calls from caregivers, while the embedded GPS helps to track seniors showing their position to the caregivers. WatchRx system also uses a patent-pending real-time predictive analytics algorithm to know if a senior is likely to miss a medication and proactively send alerts.

SmartKavach from EasyM2M [227] is an industrially designed, lightweight, rugged, waterproof device with cellular connectivity, Wi-Fi, and BLE with up to six days battery lifetime. The system includes location tracking and geo-fencing, medications reminder, fall detection, help button, video and voice calling directly connected to caregivers, and anti-theft mechanism.

Reemo watch [228] is a remote home controller which is able to operate appliances connected to sensors with specific gestures. The system collects health and wellness information which provide a clear understanding of a senior’s activity, risk factors, and behaviour changes. This information is shown on a dashboard accessible to the caregivers through computer or mobile devices, which are also alerted in case of fall detection. However, customer release has not been announced yet.

Omate announced the Wherecom S3 [229], an Android-powered smartwatch with GPS, Wi-Fi and 3G cellular connectivity, which can be used as a phone, and includes pedometer, medication reminder, and help button which sends location to pre-set contacts in case of falls. The watch also includes interchangeable straps and has up to three days battery lifetime.

Toshiba unveiled the Silmee W20 and W21 [230] both activity monitoring wristbands with skin temperature sensor, pulse monitor, ultraviolet light sensor, accelerometer and an emergency button in case of accidents or general distress. The devices also have Bluetooth capability that connects to both iOS and Android, and the W21 model has a GPS tracking system in case the wearer should become disoriented and lost. However, no further information has been provided regarding the device release.

Haier [231] unveiled at CES 2016 the senior citizens’ version of their smartwatch which is elegant and with an authentic leather strap. It shows an OLED screen, and it is waterproof down to 30 m depth with a battery life of up to two days. The built-in help button can alert up to three contact numbers in the event of an emergency. The same Android and Apple app is used for GPS localization, geo-fencing, multi-day location history storing, and has the option to retrace the wearer’s path. However, currently only the adult-version of the smartwatch is available.

Finally, the Japanese security service provider Secom [232] announced the launch of their older people-focused wristband by summer 2017 after conducting field trials. It will include a help button, fall detection, GPS localization, emergency alerts management and also fitness features (step counting, calories burned). The wristband is water-resistant and with a battery-life around 10 days.

### 5.3. Non Wrist-Related Wearables

A number of interesting elderly-oriented wearable products are available on the market. Although they are not worn on the wrist, it is worth mentioning some of them.

Amulyte [233] is a wearable activity tracker in the form of a pendant. It is lightweight and easy to use. It uses a single button to connect with caregivers in case of emergency allowing the user to speak with them through the built-in microphone and speaker. The device also transmits the location on a portal accessible remotely by the caregivers. Through its on-board accelerometer, the pendant also keeps track of the user’s activities and provides daily trends and information on the portal.

AdhereTech [234] uses a smart pill bottle with sensors and cellular technology integrated into the plastic walls of a bottle to measure medication adherence. If a patient misses a dose, the bottle reminds them with an on-bottle light, then a chime, and finally an automated text message or phone call, which can also be sent to the caregivers. AdhereTech also collects data on nonadherence, recording and tracking reasons for nonadherence. The system requires zero set-up and the battery lasts for over 200 days on a single charge.

BodyGuardian [235] is a wearable remote physiological monitoring system produced by Preventice for easy and comprehensive monitoring of a user’s cardiac activity. The lightweight sensor is discreet and pocket-size and can performs regular monitoring of ECG and cardiac rhythms. The data collected by the device can be easily accessed via a portal accessible from smartphones. In addition, BodyGuardian continuously syncs data with the Preventice CarePlatform, where healthcare professionals can monitor the wearer’s progress and results and set individualized alerts for patients, in case of irregularities detected comparing against pre-set parameters.

Regarding fall detection, a couple of products have been designed for the market. Sense4Care [236] produced Angel4, a stand-alone wearable device conceived from the excellent results achieved by the European project FATE. The device is worn on the waist and is completely automatic, without buttons or requiring any user interactions. The sensor, with its powerful algorithm having an effectiveness higher than 95%, detects any falls and automatically sends an emergency call to pre-set caregivers which, using a specific smartphone app, can localize the wearer owing to the GPS built-in in the device. The system costs $150 and has a battery lifetime of three months.

ActiveProtective [237], as an alternative approach have developed a smart clothing based system using an accelerometer that provides “fall disambiguation” determining falls prior to impact and including built-in micro-airbags to intervene for protection. Positioned on the hip of the wearer as a belt, the system protects against the consequences of falls reducing the incidence of hip fractures.

### 5.4. Wearables Systems as Healthcare Devices

As shown in face-to-face interviews with senior citizens [182], older people see the future of wearable devices in the healthcare sector by indicating the need, for stakeholders in this area, to get involved in promoting physical activity trackers to patients as a possible way to improve their health. Indeed, most of the interviewed subjects wished the devices were available in pharmacies, and that they could learn about the devices from someone in health care, such as pharmacists, similarly to what is done with other health-monitoring systems (e.g., blood glucose meters, blood pressure meters). Moreover, they were interested if doctors or other health care professionals would potentially take advantage of the data provided from the devices.

Nowadays, in Canada, activity trackers are not taxed if bought with a prescription [182], and in 2016 the PiezoRx [238], a medical-grade exercise prescription device produced by StepsCount, was recognized as a Class 1 Medical Device by Health Canada and now prescribed also by the physios’ Sports Care Centre (Ottawa’s leader in injury rehabilitation).

Similarly, in the US, a fitness tracker device is only eligible for reimbursement with a Letter of Medical Necessity (LMN) from a physician with a flexible spending account (FSA), health savings account (HSA) or a health reimbursement arrangement (HRA), in order to treat a legitimate medical condition, such as obesity [239]. The Internal Revenue Service (IRS), indeed, has ruled that fitness trackers are used to promote one’s “general health” and are only medically necessary under special circumstances.

Analogous considerations have been analyzed by the Food & Drug Association (FDA) which, in the guidance published in July 2016 [240], indicates that it does not intend to examine low-risk general wellness products, which include fitness trackers, as those products do not make a medical claim but are marketed as improving a person’s general state of health. Thus, it is evident that manufacturers can produce fitness trackers without being subject to FDA oversight, with the downside of being excluded by the healthcare service.

Therefore, it is envisaged that the future challenges and evolution for wearables are related to regulatory hurdles, compliance, and reimbursement. The current pilot programs are aiming to generate data which can validate the medical relevance of the devices and benchmark them against existing clinical solutions, so that accuracy and reliability issues would be reduced and FDA clearance can be obtained. Those medically relevant, clinical-grade datasets can initiate the integration of wearable devices into medical technologies, thus, persuading insurance companies to cover the cost of the systems for certain patients. Even though fitness trackers are not typically covered, there are some examples of wearables systems which are starting to receive some form of reimbursement, such as Body Guardian [235], Zio Patch by iRhythm [241] for remote arrhythmias detection, or the inertial-based gait analyser LEGSys by Biosensics [242], while the US Department of Veteran Affairs (VA) [243] will soon begin reimbursing for monitoring devices (such as the Leaf Patient Monitoring System for pressure ulcer prevention or the modus StepWatch activity monitoring system) that can deliver continuous and accurate data on the effectiveness of prosthetic devices.

The following section will describe in details the relationship between insurance companies and wearables.

## 6. Insurance Companies and Wearables

The evolution of wearable technology in healthcare is expected to revolutionize the health insurance industry, according to a new report from Timetric’s Insurance Intelligence Center (IIC) [244].

According to a study conducted in 2014 by Strategy Meets Action (SMA) [245], a Boston-based research advisory firm for the insurance industry, 3% of insurers were already making use of wearable devices with nearly 22% of them in the process of developing a strategy to deploy them.

Moreover, in the Wearable Future report written in 2014 by PwC [246] it was reported that 68% of consumers would wear employer-provided wearables streaming anonymous data to an information pool in exchange for break on their insurance premiums. Furthermore, consumers were more willing to try wearable technology provided by their primary care doctor’s office than they are from any other brand or category.

A survey [247] in Accenture’s Technology Vision Report in 2015 across nine countries has shown that 63% of the insurer executives believed wearables will be adopted broadly by the insurance industry within the next two years, while 31% said they were already using wearables to engage customers, employees or partners, and the 51% of insurers planned to partner with major digital technology and cloud platform leaders in the next two years.

Finally, the Insurance Barometer Study [248], produced in 2016 by Life Happens in collaboration with LIMRA, showed that 51% of Millennials and 30% of people overall were very or extremely likely to consider wearing an activity tracker and share those results with a life insurance company in return for financial rewards for healthy behaviours. The number went up to 65% when considering consumers who already used an activity tracker. The results revealed that more than a quarter of Americans (27%) and a third of Millennials (33%) cite the potential to build a long-term relationship with an insurance company as a reason to share biometric data from a wearable activity tracker.

It is, therefore, not surprising that, according to EY’s 2016 Sensor Data Survey of senior executives from nearly 400 insurers globally, wearable sensors will be one of the most important data sources for future competitiveness within their industry [249].

Wearables are now providing holistic and wellness-focused analytics, which can play a crucial role in several aspects of individual and group insurance plans, given their ability to provide relevant data and genuine insight in regards to the actual behavioural patterns. More specifically, insurers can use this information to set targets and incentives for policyholders to live a healthier lifestyle, encouraging them with the possibility of lower premiums, following a Pay-As-You-Live (PAYL) scheme. Insurance companies can, thus, greatly benefit by integrating wearable technology into their value chain. As discussed in Reference [250,251,252], they can expect potential use in marketing, underwriting, risk management, product development, compensation and claims management, such as:

- *Customer engagement*: Support customer health and wellness, encouraging and rewarding healthy behaviours with financial incentives or lower premiums, while reducing risk for life and health insurance by decreasing the frequency and size of claims. Notification of customers in the event of a sudden adverse health condition is also a possibility. Predictive analytics can also be used to project future potential threat/abnormality in insured customers, warning them in real-time of the impending risks. Changes in policyholder details are detected and updated according to the collected data.
- *Risk assessment*: Help insurers better understand their customers by creating individual biometric profiles and identifying customer segments that can be used to deliver personalized targeted products and services.
- *Collaboration with healthcare providers*: Create growth opportunities by building an ecosystem of partnerships (such as health and wellness loyalty programs tied to wearable use) to access to new customers. Partnership between health insurers and healthcare service providers. Monitoring and managing recuperation time of patients, further helping claims management.
- *Customized products and services*: Improve retention by leveraging wearable data analytics to create personalised customer propositions. Launch new product models and flexible options with bonus/penalty characteristics (in case of overachieving/not meeting set targets) and with premium pricing to policyholders sharing health reports and personal data, for better management of insurance float.
- *Savings*: Reduce the cost of customer on-boarding and identification, and the cost of providing healthcare. Identify and prevent fraudulent activities, lowering claims expenses and increasing customer loyalty, and beginning of new real-time claims management by the adjuster.

Nowadays, 60% of European top insurers have launched connected car solutions using smartphone apps, dongles, or black-box devices, and it is expected that connected health solutions will experience a similar growth [253], also pushed by employers integrating wearables into employees’ wellness programs [254]. Some examples of the insurance companies proceeding along this pathway are discussed below.

John Hancock [255], an American-based life insurer, with its Vitality Program offers its policyholders Fitbits to track wellness goals and receive discounts up to 15% on life insurance annual premium for hitting targets, up to $600 in annual savings on healthy food purchases, and discounts and rewards from several companies.

In September 2014, Oscar Health Insurance [256], a New York insurance company, started supplying customers with a Misfit Flash and offered users $1 credit per day when they hit their personalized step goal, with rewards in Amazon vouchers of up to $20 per month.

Prudential’s Vitality Health [257] offers its life insurance customers points that can be spent on rewards such as cinema tickets, and discounted spa days, alongside reduced premiums. Those points are earned when specific targets are met. Discounts for purchasing an Apple Watch are also recently available with this program.

AIA Australia [258], a life insurance company, through its Vitality program offers to members savings up to 25% off the recommended retail price of several fitness trackers and earn AIA Vitality Points when using them.

Medibank [259], a private health insurer in Australia, allows members to save 25% on a range of Fitbit activity trackers.

AXA [260] partnered with Withings to offer to AXA customers a free Withings Pulse as well as the possibility of getting discounts on medications and future Withings purchases, if they walk 7000+ or 10,000+ steps per day for a period of one month.

MLC in Australia defined a new wellness program, MLC Life Insurance on Track [261], where members adopt a Garmin Vivosmart HR tracker to receive up to 10% off the life insurance premium if wellness scores related to steps, sleep time, and heart rate are met. A similar strategy was pursued by South African health insurer Discovery Health [262] with its Vitality program and savings up to 900 points per week for funding an Apple Watch.

Similarly, Manulife Financial Corp. in Canada [263] provides, as part of its Vitality program, a Garmin Vivofit 3 to their life insurance members, rewarding policyholders when a certain numbers of points are accumulated.

Highmark [264] has teamed up with Walkadoo to provide a low-cost activity tracking program for health plan members. Members receive a Smart Step Pebble and a lifetime online membership in Walkadoo, earning points according to the amount of activities completed daily.

South African insurer Momentum [265], with its program multiply, rewards members with discounts and returns on a variety of products when reaching pre-set goals measured by an activity tracker. An analogous program was recently adopted also by the Chinese health insurer Ping An with its Vitality [266].

Blue Cross Blue Shield Associated released a Blue365 program [267] to provide discounts for members to a series of devices promoting health, fitness, and personal care, such as sleep trackers, Garmin, Sensoria, Lively, etc.…

Cigna, with its Silver & Fit program [268], motivate and encourage older adults rewarding them for being active, tracking the exercises with fitness device or apps.

Finally, also Aetna [269], in September 2016, has declared they will be launching a wellness program which includes an Apple Watch.

However, many other companies launched products for employers willing to include wearables in employees’ wellness programs. Some examples include:

- Connecticut-based life insurer Phoenix [270] donated 300 fitness trackers to employees for a step-tracking competition;
- US-based Appirio [271] negotiated a 5% discount on the health insurance bill by sharing data collected by employees with Fitbit^®^;
- Self-insured BP America [272] donated Fitbit^®^ Zips to 14,000 employees with the possibility to lower insurance premium if meeting the goal of one million steps counted;
- Insurer UnitedHealthcare Motion and Qualcomm [273] partnered to provide complimentary Fitbits^®^ or Jawbones to Qualcomm employees, who are able to earn up to $1460 when meeting certain goals;
- Allianz is promoting the use of dorsaVi [274], a leader in motion technology providing wearable sensors, to reduce injuries to workers, reduce occupational health and safety costs for employers, manage fewer claims on insurers, and help employers manage the cost of their insurance premiums;
- Health insurance provider Humana [275] launched Go365 to provide personalized wellness and reward program for employees, including diet and nutrition apps, activity trackers and sleep monitors. In a 3-year impact study, it was reported that this program can lead to lower health claim costs, less absenteeism, less emergency healthcare consumption, and fewer lifestyle risk factors for chronic disease.

Despite the number of advantages, the adoption of wearable technologies by the insurance industry presents some challenges. Privacy is a major issue for most policyholders, who have to decide which parameters can be shared with the insurance company. Moreover, current benefits are still not sufficient to motivate people in wearing an activity tracker, causing low opt-in rates. A consequence of the lack of customer base involves the presence of insufficient customer data useful for building reliable predictive analytics and new products. Therefore, gamification is another evident trend which is useful for engaging customers to increase their level of motivation and compliance.

Insurers have to build trust with customers by relying on complex customer engagement strategies. Insurers also need to build a solid framework to collect unstructured data, securely store them, and analyse them. Such infrastructures will require high investment for connected information, for implementing a multi-platform structure covering different wearable models, and also to guarantee a high level of security against potential breaches.

The lack of standards around user privacy and security issues can be a potential regulatory hurdle as well as the low integration between public and private healthcare systems.

Additionally, technology providers should increase the reliability of their products, by limiting technology errors, malfunctioning devices, improving connectivity, and enhancing the analytics for better healthcare delivery and treatments. Current devices have different specifications, thus providing inconsistent quality of data precision. Moreover, given the impact that wearables and activity data will have on insurance premiums, future devices should be able to recognize ‘real’ activities performed by the policyholders from potentially ‘simulated’ movements performed by third-parties.

Finally, an ethical issue may revolve around the inclusion, in those insurance-defined wearable-based new products, of people with specific conditions (e.g., subjects on a wheelchair), which, in programs based solely on physical activity evaluation, may be disadvantaged by earning a lower amount of points/discounts compared to normal subjects owing to their conditions.

The technology is advancing rapidly and the market for wearable technology will expand significantly. Despite potential restraints and barriers, such data can cause a dramatic shift in the future life and health insurance industry.

## 7. Conclusions

Chronic and ageing-related diseases are affecting a great amount of resources in western countries healthcare systems. Even though they can be prevented by increasing the level of an individual’s Physical Activity (PA), an objective and accurate PA assessment is still an issue. Among the several devices considered to this purpose, it has been reported that wearable motion detectors are the most promising technology enabling an automatic, continuous and long-term assessment of subjects in free-living environments. This topic is so current that a search on ‘wearable sensors for elderly people’ on Google Scholar, listed over 1800 results since 2017 including [276,277,278].

Several approaches aiming to provide posture detection and state transitions estimation, gait analysis, balance assessment, fall detection and related risk assessment in elderly subjects through accelerometers-based sensors have been thoroughly described, together with the application of commercial fitness trackers in the senior citizens population. Their validity and acceptability reported in a number of studies in clinical literature has been analysed alongside a discussion related to the features required for the population taken into account.

Moreover, an in-depth analysis of the current market landscape related to activity trackers tailored for older adults was described, followed by an outline depiction of the potential future trends associated to regulatory hurdles and reimbursements.

Finally, the impact of wearables on life and health insurance companies was shown in details with several examples of the potential benefits for the industry and the wearable market.

In the future, the adoption of wearable inertial-based activity monitors by older people should be tailored by tracker manufacturers. Obtained physical activity parameters can be shared with healthcare providers and insurance platforms to better describe behavioural pattern and functional ability in high-risk subjects, thus providing important feedback regarding the overall health status of an individual and even prediction of potential adverse health events.

## Figures and Tables

**Table 1:** Remote Monitoring Systems for Senior Citizens.

Reference	Sensors	Placement	Methods	Measurement Context	Final Report
Noury [45]	Accelerometer, Tilt Switch, Vibration Sensor	N/A	Activity and Fall Detection	Lab (1 subject)	Data sent wirelessly to a PC
Noury et al. [46]	3D Accelerometer, 3D Magnetometer	Chest, Dominant Wrist, Thigh, Ankle	Activity and Transition Estimation	Lab and Clinical (5 subjects)	N/A
Bourke et al. [47]	3D Accelerometer	Trunk	Fall Detection	Lab (11 subjects)	N/A
Prado et al. [48]	Accelerometer	Sacrum	Activity and Fall Detection	Lab (N/A)	N/A
Miyauchi et al. [49]	Accelerometer, Mobile Phone with GPS	Abdomen	Fall Detection	Lab (1 subject)	Fall events are transmitted from the phone to a PC which sends subject’s location to caregivers
Dinh et al. [50]	3D Accelerometer, 2D Gyro, Heart Rate	Chest	Vital Sign Monitoring and Fall Detection	N/A	Data are sent via remote network to healthcare personnel
Yazaki et al. [51]	3D Accelerometer, Temperature, ECG	Chest	Vital Sign Monitoring and Fall Detection	Lab (8 subjects)	Data are sent via remote network to family if abnormalities are detected
Pioggia et al. [52]	3D Accelerometers, ECG, Breath Rate, sEMG	Arms, Chest, Hip, Thighs	Movement Analysis and Muscle Fatigue Detection	Clinical (58 subjects)	Data sent wirelessly to a PC
Bourke et al. [53]	3D Accelerometer, Heart Rate, Respiratory Rate, Temperature	Trunk	Activity Detection, Fall Detection, Energy Expenditure	Lab (8 subjects), Clinical (9 subjects)	Data are sent via remote network to healthcare personnel
Xu et al. [54]	3D Accelerometer, Pulse Sensor, Pressure Sensors	Head, Wrists, Ankles	Fall Detection	N/A	Data sent wirelessly to a PC
Maki et al. [55]	3D Accelerometer	Chest	Vital Sign Monitoring and Activity Detection	Lab (5 subjects)	Data are sent via remote network to caregivers
Carus et al. [56]	3D Accelerometer	Wrist	Activity Detection	Real-Life (3 subjects)	Data are sent via remote network to caregivers and family if abnormalities are detected
John et al. [57]	3D Accelerometer	Waist	Energy Expenditure	Real-Life (5 subjects)	N/A
Dong et al. [58]	3D Accelerometer, 3D Gyro, Mobile Phone	Wrist	Activity Detection	N/A	Data are sent via remote network to caregivers
Terroso et al. [59]	3D Accelerometer, Mobile Phone with GPS	Chest	Fall Detection	Lab (N/A)	Data are sent via remote network to family if fall detected
Ghazal et al. [60]	3D Accelerometer, 3D Gyroscope	Wrist	Fall detection, Health Journal, Food Recommendation	Lab (N/A)	Data are sent via remote network to caregivers
Panicker et al. [61]	3D Accelerometer, Mobile Phone with GPS	N/A	Fall Detection	N/A	Data are sent via remote network to caregivers if fall detected
Srisuphab et al. [62]	3D Accelerometer, Mobile Phone with GPS	Pocket	Fall Detection	Lab (5 subjects)	Data are sent via cloud-based network to caregivers
Sriborrirux et al. [63]	3D Accelerometer	Necklace	Fall Detection, Activity Detection, Energy Expenditure	Hospital (20 subjects)	Alerts are sent wiressly to a watch worn by caregivers

**Table 2:** Activity Classification Algorithms for Senior Citizens.

Reference	Sensors	Placement	Methodology	Activities Considered	Measurement Context	Accuracy (%)
Najafi et al. [102]	Two 2D Accelerometers, 1D Gyro	Chest	Discrete Wavelet Transform	Sitting, Standing, Lying, Walking	Lab (11 subjects), Clinical (24 subjects), Real-Life (9 subjects)	Sensitivity: 93.6; Specificity: 95.1
Culhane et al. [103]	Two 2D Accelerometers	Thigh, Chest	Threshold-based	Sitting, Standing, Lying	Clinical (5 subjects)	92
Paiyarom et al. [104]	3D Accelerometer	Waist	Dynamic Time Warping	Standing, Sitting, Transitions Lying, Walking, Running	Lab (2 subjects)	91
Kang et al. [105]	3D accelerometer	Waist	Hierarchical Binary Tree	Standing, Sitting, Transitions Lying, Walking, Running, Falling	Lab (5 subjects)	96.1
Khan et al. [106]	3D Accelerometer	Chest/Trouser Pockets	Artificial Neural Networks + Linear Discriminant Analysis	Sitting, Standing, Lying, Walking (up/down), Running, Cycling, Vacuuming	Real-Life (8 subjects)	94
Sekine et al. [107]	3D Accelerometer	Waist	Discrete Wavelet Transform	Walking (up/down)	Lab (11 subjects)	N/A
Muscillo et al. [108]	2D Accelerometer	Shin	Artificial Neural Networks + Kalman Filters	Walking (up/down)	Lab (24 subjects)	92
Weiss et al. [109]	3D Accelerometer, 3D Gyro	Lower Back	Sensors-Derived Measures	Walking (up/down)	Lab (17 subjects)	N/A
Chernbumroong et al. [110]	Heart Rate, 3D Accelerometers, 3D Gyro, Light Sensor, Barometer, Temperature, Altimeter	Chest, Wrists	Genetic Algorithm + Neural Networks and SVM	Household Activities	Lab (12 subjects)	98
Ul Alam et al. [111]	EDA, PPG, 3D Accelerometer	Wrist	Machine Learning	Household Activities	Community (17 subjects)	92
Sasaki et al. [112]	Three 3D Accelerometers	Hip, Wrist, Ankle	Random Forest + SVM	Standing, Sitting, Lying, Walking, Household/Recreational Activities	Lab/Real-Life (35 subjects)	55/69
Papadopoulos et al. [113]	3D Accelerometer	Wrist	Time/Frequency Measures	Walking vs Other Activities	Real-Life (30 subjects)	98

**Table 3:** Gait Analysis Systems for Senior Citizens.

Reference	Sensors	Placement	Parameters of Interest	Measurement Context/Participants/Age	Test/Validation	Accuracy and/or Other Details
Mariani et al. [120]	3D Accelerometer, 3D Gyro	Foot	Stride Length, Foot Clearance, Turning Angle, Stride Velocity	Lab/10 young subjects, 10 elderly/26.1 and 71.6	U-shaped, 8-shaped trials and 6MWT/VICON	Mean Error: 1.5 cm, 1.9 cm, 1.6°, 1.4 cm/s
Rampp et al. [121]	3D Accelerometer, 3D Gyro	Foot	Stride Length, Stride Time, Swing Time, Stance Time	Clinical/116 subjects/82.1	10 m walking/GAITRite	6.26 cm on stride length on normal walking
Dadashi et al. [122]	3D Accelerometer, 3D Gyro	Foot	Time-Spatial Parameters, Heel/Toe Clearance	Clinical/1400 subjects/>65	20 m walking	Significant difference between men and women
Zhang et al. [123]	3D Accelerometer	Ankle	Step Frequency, Gait Duration	Real-Life/297 subjects/65.7	Unconstrained daily activities for 7 days	ICC between 0.668 and 0.873
Atallah et al. [124]	3D Accelerometer	Ear	Gait Cycle Time, Step Asymmetry	Lab/64 subjects/60.04	Walking test/Instrumented treadmill	Mean difference 10 ms
Takenoshita et al. [125]	3D Accelerometer	Lower Back	Walking Speed, Centre of Gravity	Clinical/402 subjects/78.2	Walking test for 3 months	Walking speed decreases with time in clinic
Chan et al. [126]	3D Accelerometer, 3D Gyro	Lower Back	Cadence, Stride/Step Regularity, Symmetry used as Features	Lab/ 13 young subjects, 12 elderly/27.7 and 70	Walking up/downstairs	Discriminate between young and elderly subjects (95.7%)
Clermont et al. [127]	3D Accelerometer	Lower Back	Speed Time, Step Time, Stride Time	Lab/30 subjects/65.32	200 m walking test	Higher stride/step time for subjects with knee osteoarthritis
Del Din et al. [128]	3D Accelerometer	Lower Back	Time-Spatial Parameters, Variability, Asymmetry	Lab/60 subjects/66.75	10 m walking/GAITRite	ICC between 0.913 and 0.983 for 4 gait characteristics
Hartmann et al. [129]	3D Accelerometer	Lower Back	Time-Spatial Parameters and Variability	Lab/23 subjects/77.2	10 m walking/GAITRite	High ICCs between 0.99 and 1 for averaged step data
Hartmann et al. [130]	3D Accelerometer	Lower Back	Time-Spatial Parameters and Variability	Lab/23 subjects/73.4	Walking test on different surfaces	ICC for speed, cadence, step time and step length on different surfaces and dual-task conditions
Grimpampi et al. [131]	3D Accelerometer, 3D Gyro	Lower Trunk	Time-Spatial Parameters and Variability	Lab/29 subjects/84	6MWT	High ICCs between 0.93 and 0.95 for all parameters
Donath et al. [132]	3D Accelerometer, 3D Gyro, 3D Magnetometer	Foot	Time-Spatial Parameters	Lab/24 subjects/75.3	Walking test/Instrumented treadmill	ICCs between 0.99 and 1 for time variables, except for stride length at low speed
Brodie et al. [134]	3D Accelerometer, Barometer	Pendant	Cadence, Speed, Stride length, Step Time Variability	Real-Life and Lab/51 subjects/83	Unconstrained daily activities/Video and walkway	Step time variability is higher and uncorrelated with lab-assessed results

## References

[1] Population Division, Department of Economic and Social Affairs, United Nations World Population Ageing 2015. http://www.un.org/en/development/desa/population/publications/pdf/ageing/WPA2015_Report.pdf.

[2] World Health Organization The World Health Report 2002—Reducing Risks, Promoting Healthy Life. http://www.who.int/whr/2002/en/.

[3] Willett W.C., Koplan J.P., Nugent R., Dusenbury C., Puska P., Gaziano T.A., Jamison D.T., Breman J.G., Measham A.R. (2006). Prevention of chronic disease by means of diet and lifestyle changes. Disease Control Priorities in Developing Countries.

[4] O’Flynn B., Torres J., Angove P., Connolly J., Condell J., Curran K., Gardiner P. Novel smart sensor glove for arthritis rehabilitation. Proceedings of the IEEE International Conference on Body Sensor Networks (BSN).

[5] Walsh M., Barton J., O’Flynn B., O’Mathuna C., Hickey A., Kellett J. On the relationship between cummulative movement, clinical scores and clinical outcomes. Proceedings of the IEEE Sensors.

[6] Walsh M., O’Flynn B., O’Mathuna C., Hickey A., Kellett J., O’Grady M.J. (2013). Correlating average cumulative movement and Barthel Index in acute elderly care. Evolving Ambient Intelligence. AmI 2013. Communications in Computer and Information Science.

[7] Tedesco S., Urru A., Peckitt J., O’Flynn B. Wearable inertial sensors as a tool for quantitative assessment of progress during rehabilitation. Proceedings of the International Conference on Global Health Challenges.

[8] Sedentary Behaviour Research Network (2012). Letter to the editor: Standardized use of the terms “sedentary” and “sedentary behaviours”. Appl. Physiol. Nutr. Metab..

[9] Bouchard C., Shephard R.J., Stephens T. (1994). Physical Activity, Fitness, and Health: Consensus Statement.

[10] Van der Ploeg H.P., Chey T., Korda R.J., Banks E., Bauman A. (2012). Sitting time and all-cause mortality risk in 222,497 Australian adults. JAMA Intern. Med..

[11] Hills A.P., Mokhtar N., Byrne N.M. (2014). Assessment of physical activity and energy expenditure: An overview of objective measures. Front. Nutr..

[12] Matthews C.E., Hagströmer M., Pober D.M., Bowles H.R. (2012). Best practices for using physical activity monitors in population-based research. Med. Sci. Sports Exerc..

[13] Morrison S., Colberg S.R., Parson H.K., Neumann S., Handel R., Vinik E.J., Paulson J., Vinik A.I. (2016). Walking-induced fatigue leads to increased falls risk in older adults. J. Am. Med. Dir. Assoc..

[14] Yang C.C., Hsu Y.L. (2010). A review of accelerometry-based wearable motion detectors for physical activity monitoring. Sensors.

[15] Igual R., Medrano C., Plaza I. (2013). Challenges, issues and trends in fall detection systems. Biomed. Eng. Online.

[16] Salzman B. (2010). Gait and balance disorders in older adults. Am. Fam. Phys..

[17] Moraes W., Piovezan R., Poyares D., Bittencourt L.R., Santos-Silva R., Tufik S. (2014). Effects of aging on sleep structure throughout adulthood: A population-based study. Sleep Med..

[18] Nam Y., Kim Y., Lee J. (2016). Sleep monitoring based on a tri-axial accelerometer and a pressure sensor. Sensors.

[19] Ong A.A., Gillespie M.B. (2016). Overview of smartphone applications for sleep analysis. World J. Otorhinolaryngol. Head Neck Surg..

[20] Roebuck A., Monasterio V., Gederi E., Osipov M., Behar J., Malhotra A., Penzel T., Clifford G.D. (2014). A review of signals used in sleep analysis. Physiol. Meas..

[21] Gupta L., Morgan K., Gilchrist S. (2016). Does elite sport degrade sleep quality? A systematic review. Sports Med..

[22] Silva A.A.D., Mello R.G.B.D., Schaan C.W., Fuchs F.D., Redline S., Fuchs S.C. (2016). Sleep duration and mortality in the elderly: A systematic review with meta-analysis. BMJ Open.

[23] Loveday A., Sherar L.B., Sanders J.P., Sanderson P.W., Esliger D.W. (2015). Technologies that assess the location of physical activity and sedentary behavior: A systematic review. J. Med. Internet Res..

[24] Bauman A., Phongsvan P., Schoeppe S., Owen N. (2006). Physical activity measurement—A primer for health promotion. Promot. Educ..

[25] Liao S.Y., Benzo R., Ries A.L., Soler X. (2014). Physical activity monitoring in patients with chronic obstructive pulmonary disease. J. COPD Found..

[26] Fiore L., Fehr D., Bodor R., Drenner A., Somasundaram G., Papanikolopoulos N. (2008). Multi-camera human activity monitoring. J. Intell. Robot. Syst..

[27] Ozcan K., Mahabalagiri A., Velipasalar S., Bobda C., Velipasalar S. (2014). Automatic fall detection and activity classification by a wearable camera. Distributed Embedded Smart Cameras: Architectures, Design and Applications.

[28] Demiris G., Thompson H. (2011). Smart homes and ambient assisted living applications: From data to knowledge-empowering or overwhelming older adults? Contribution of the IMIA Smart Homes and Ambiant Assisted Living Working Group. Yearb. Med. Inform..

[29] Schrack J.A., Zipunnikov V., Goldsmith J., Bandeen-Roche K., Crainiceanu C.M., Ferrucci L. (2014). Estimating energy expenditure from heart rate in older adults: A case for calibration. PLoS ONE.

[30] Kushida C.A., Littner M.R., Morgenthaler T., Alessi C.A., Bailey D., Coleman J., Friedman L., Hirshkowitz M., Kapen S., Kramer M. (2005). Practice parameters for the indications for polysomnography and related procedures: An update for 2005. Sleep.

[31] Beevi F.H., Miranda J., Pedersen C.F., Wagner S. (2016). An evaluation of commercial pedometers for monitoring slow walking speed populations. Telemed. e-Health.

[32] Kamada M., Shiroma E.J., Harris T.B., Lee I.M. (2016). Comparison of physical activity assessed using hip- and wrist-worn accelerometers. Gait Posture.

[33] Parkka J., Ermes M., Antila K., van Gils M., Manttari A., Nieminen H. Estimating intensity of physical activity: A comparison of wearable accelerometer and gyro sensors and 3 sensor locations. Proceedings of the IEEE International Conference of Engineering in Medicine and Biology Society (EMBS).

[34] Rodriguez-Martin D., Perez-Lopez C., Sama A., Cabestany J., Catala A. (2013). A wearable inertial measurement unit for long-term monitoring in the dependency care area. Sensors.

[35] Shoaib M., Bosch S., Incel O.D., Scholten H., Havinga P.J.M. (2014). Fusion of smartphone motion sensors for physical activity recognition. Sensors.

[36] Chen K.Y., Janz K.F., Zhu W., Brychta R.J. (2012). Re-defining the roles of sensors in objective physical activity monitoring. Med. Sci. Sports Exerc..

[37] Choudhary V., Iniewski K. (2013). MEMS Fundamental Technology and Applications.

[38] Ciuti G., Ricotti L., Menciassi A., Dario P. (2015). MEMS sensor technologies for human centred applications in healthcare, physical activities, safety and environmental sensing: A review on research activities in Italy. Sensors.

[39] Del Rosario M.B., Redmond S.J., Lovell N.H. (2015). Tracking the evolution of smartphone sensing for monitoring human movement. Sensors.

[40] Freedson P., Bowles H.R., Troiano R., Haskell W. (2012). Assessment of physical activity using wearable monitors: Recommendations for monitor calibration and use in the field. Med. Sci. Sports Exerc..

[41] Taraldsen K., Chastin S.F., Riphagenc I.I., Vereijkend B., Helbostad J.L. (2012). Physical activity monitoring by use of accelerometer-based body-worn sensors in older adults: A systematic literature review of current knowledge and applications. Maturitas.

[42] De Bruin E.D., Hartmann A., Uebelhart D., Murer K., Zijlstra W. (2008). Wearable systems for monitoring mobility-related activities in older people: A systematic review. Clin. Rehabil..

[43] Santiago Delahoz Y., Labrador M.A. (2014). Survey on fall detection and fall prevention using wearable and external sensors. Sensors.

[44] Attal F., Mohammed S., Dedabrishvili M., Chamroukhi F., Oukhellou L., Amirat Y. (2015). Physical human activity recognition using wearable sensors. Sensors.

[45] Noury N. A smart sensor for the remote follow up of activity and fall detection of the elderly. Proceedings of the Annual International IEEE-EMBS Special Topic Conference on Microtechnologies in Medicine & Biology.

[46] Noury N., Barralon P., Couturier P., Favre-Reguillon F., Guillemaud R., Mestais C., Caritu Y., David D., Moine S., Franco A. ACTIDOM—A microsystem based on MEMS for activity monitoring of the frail elderly in their daily life. Proceedings of the IEEE International Conference of Engineering in Medicine and Biology Society (EMBS).

[47] Bourke A.K., van de Ven P.W., Chaya A.E., ÓLaighin G.M., Nelson J. The design and development of a long-term fall detection system incorporated into a custom vest for the elderly. Proceedings of the IEEE International Conference of Engineering in Medicine and Biology Society (EMBS).

[48] Prado M., Reina-Tosina J., Roa L. Distributed intelligent architecture for falling detection and physical activity analysis in the elderly. Proceedings of the Joint Annual Conference and the Annual Fall Meeting of the Biomedical Engineering Society EMBS/BMES.

[49] Miyauchi K., Yonezawa Y., Ogawa H., Maki H., Caldwell W.M. A mobile phone-based safety and life support system for elderly people. Proceedings of the IEEE Conference on Consumer Communications and Networking.

[50] Dinh A., Teng D., Chen L., Shi Y., McCrosky C., Basran J., Del Bello-Haas V. Implementation of a physical activity monitoring system for the elderly people with built-in vital sign and fall detection. Proceedings of the International Conference on Information Technology: New Generations.

[51] Yazaki S., Matsunaga T. Portable life support system using wearable biosensor worn by the elderly. Proceedings of the International Conference on ICROS-SICE.

[52] Pioggia G., Tartarisco G., Valenza G., Ricci G., Volpi L., Siciliano G., Bonfiglio S. A pervasive activity management and rehabilitation support system for the elderly. Proceedings of the International Conference on Pervasive Computing and Communications Workshops (PERCOM).

[53] Bourke A.K., Prescher S., Koelher F., Cionca V., Tavares C., Gomis S., Garcia V., Nelson J. Embedded fall and activity monitoring for a wearable ambient assisted living solution for older adults. Proceedings of the International Conference of Engineering in Medicine and Biology Society (EMBC).

[54] Xu G., Li J., Wang L., Wu J., Luo K., Yue Y., Ran F. Hardware design of A body sensor network system used for elder care. Proceedings of the International Conference on Natural Computation (ICNC).

[55] Maki H., Ogawa H., Matsuoka S., Yonezawa Y., Caldwell W.M. A daily living activity remote monitoring system for solitary elderly people. Proceedings of the International Conference of Engineering in Medicine and Biology Society (EMBC).

[56] Carus J.L., Pelaez V., Garcia S., Fernandez M.A., Diaz G., Alvarez E. A non-invasive and autonomous physical activity measurement system for the elderly. Proceedings of the IEEE International Conference on Ubiquitous Intelligence & Computing and Autonomic & Trusted Computing.

[57] John S.G., Owen P.J., Smith K., Youde J.H., McIntyre C.W. Utilisation of telemedicine to assess energy expenditure and stability in older people with chronic kidney disease. Proceedings of the Computers in Cardiology.

[58] Dong M., Hou Z., Liu Z., Bi S., Lin P., Zhu L., Xin J., Liu L. Design and implementation of behaviour detection system for the elderly based on smart phone. Proceedings of the IEEE International Conference on Robotics and Biomimetics (ROBIO).

[59] Terroso M., Freitas R., Gabriel J., Torres Marques A., Simoes R. Active assistance for senior healthcare: A wearable system for fall detection. Proceedings of the Iberian Conference on Information Systems Technologies (CISTI).

[60] Ghazal M., Al Khalil Y., Dehbozorgi F.J., Alhalabi M.T. An integrated caregiver-focused mHealth framework for elderly care. Proceedings of the IEEE International Conference on Wireless and Mobile Computing, Networking, and Communications.

[61] Panicker N.V., Kumar S.A. Design of a telemonitoring system for detecting falls of the elderly. Proceedings of the International Conference on Green Computing and Internet of Things (ICGIoT).

[62] Srisuphab A., Silapachote P., Phongpawarit J., Visetpalitpol S., Jirapasitchai S. REDLE: Elderly care on clouds. Proceedings of the International Conference on Computer Science and Software Engineering (JCSSE).

[63] Sriborrirux W., Leamsumran P., Dan-klang P. Real-time system for monitoring activity among the elderly using an RF SoC device with triaxial accelerometer data over a wireless sensor network. Proceedings of the IEEE MTT-S International Microwave Workshop Series on RF and Wireless Technologies for Biomedical and Healthcare Applications (IMWS-Bio).

[64] Sallis J.F. (2000). Age-related decline in physical activity: A synthesis of human and animal studies. Med. Sci. Sports Exerc..

[65] Murphy S.L. (2009). Review of physical activity measurement using accelerometers in older adults: Considerations for research design and conduct. Prev. Med..

[66] ActiGraph Corp. http://actigraphcorp.com/.

[67] Actiwatch Spectrum. http://www.usa.philips.com/healthcare/product/HC1046964/actiwatch-spectrum-activity-monitor.

[68] PALtechnologies. http://www.paltechnologies.com/products/.

[69] Dynaport McRoberts. https://www.mcroberts.nl/old/products/movemonitor/dynaport.

[70] Ortlieb S., Dias A., Gorzelniak L., Nowak D., Karrasch S., Peters A., Kuhn K.A., Horsch A., Schulz H., KORA Study Group (2014). Exploring patterns of accelerometry-assessed physical activity in elderly people. Int. J. Behav. Nutr. Phys. Act..

[71] Shiroma E.J., Freedson P.S., Trost S.G., Lee I.M. (2013). Patterns of accelerometer-assessed sedentary behavior in older women. JAMA.

[72] Lindamer L.A., McKibbin C., Norman G.J., Jordan L., Harrison K., Abeyesinhe S., Patrick K. (2008). Assessment of physical activity in middle-ages and older adults with schizophrenia. Schizophr. Res..

[73] Klaren R.E., Sebastiao E., Chiu C.Y., Kinnett-Hopkins D., McAuley E., Motl R.W. (2016). Level and rates of physical activity in older adults with multiple sclerosis. Aging Dis..

[74] Hollewand A.M., Spijkerman A.G., Bilo H.J., Kleefstra N., Kamsma Y., van Hateren K.J. (2016). Validity of an accelerometer-based activity monitor system for measuring physical activity in frail older adults. J. Aging Phys. Act..

[75] Casey C.M., Bennett J.A., Winters-Stone K., Knafl G.J., Young H.M. (2014). Measuring activity levels associated with rehabilitative care in hospitalized older adults. Geriatr. Nurs..

[76] Ong T., Anand V., Tan W., Watson A., Sahota O. (2016). Physical activity study of older people in hospital: A cross-sectional analysis using accelerometers. Eur. Geriatr. Med..

[77] Tang Y., Green P., Maurer M., Lazarte R., Kuzniecky J.R., Hung M.Y., Garcia M., Kodali S., Harris T. (2015). Relationship between accelerometer-measured activity and self-reported or performance-based function in older adults with severe aortic stenosis. Curr. Geriatr. Rep..

[78] Narai E., Hagino H., Komatsu T., Togo F. (2016). Accelerometer-based monitoring of upper limb movement in older adults with acute and subacute stroke. J. Geriatr. Phys. Ther..

[79] Parsons T.J., Sartini C., Ellins E.A., Halcox J.P.J., Smith K.E., Ash S., Lennon L.T., Wannamethee S.G., Lee I.M., Whincup P.H. (2016). Objectively measured physical activity, sedentary time and subclinical vascular disease: Cross-sectional study in older British men. Prev. Med..

[80] Huisingh-Scheetz M.J., Kocherginsky M., Magett E., Rush P., Dale W., Waite L. (2016). Relating wrist accelerometry measures to disabilities in older adults. Arch. Gerontol. Geriatr..

[81] Dunlop D.D., Song J., Arnston E.K., Semanik P.A., Lee J., Chang R.W., Hootman J.M. (2015). Sedentary time in US older adults associated with disability in activities of daily living independent of physical activity. J. Phys. Act. Health.

[82] Johnson L.G., Butson M.L., Polman R.C., Raj I.S., Borkoles E., Scott D., Aitken D., Jones G. (2016). Light physical activity is positively associated with cognitive performance in older community dwelling adults. J. Sci. Med. Sport.

[83] Kerr J., Marshall S.J., Patterson R.E., Marinac C.R., Natarajan L., Rosenberg D., Wasilenko K., Crist K. (2013). Objectively measured physical activity and cognitive function in older adults. J. Am. Geriatr. Soc..

[84] Chase J.M., Lockhart C.K., Ashe M.C., Madden K.M. (2014). Accelerometer-based measures of sedentary behavior and cardio-metabolic risk in active older adults. Clin. Investig. Med..

[85] Gerdhem P., Dencker M., Ringsberg K., Akesson K. (2008). Accelerometer-measured daily physical activity among octogenarians: Results and associations to other indices of physical performance and bone density. Eur. J. Appl. Physiol..

[86] Foong Y.C., Chherawala N., Aitken D., Scott D., Winzenberg T., Jones G. (2016). Accelerometer-determined physical activity, muscle mass, and leg strength in community-dwelling older adults. J. Cachexia Sarcopenia Muscle.

[87] Yoshiuchi K., Inada S., Nakahara R., Akabayashi A., Park H., Park S., Shephard R.J., Aoyagi Y. (2010). Stressful life events and habitual physical activity in older adults: 1-year accelerometer data from the Nakanojo study. Mental Health Phys. Act..

[88] Godfrey A., Lord S., Galna B., Mathers J.C., Burn D.J., Rochester L. (2014). The association between retirement and age on physical activity in older adults. Age Ageing.

[89] Lohne-Seiler H., Hansen B.H., Kolle E., Anderssen S.A. (2014). Accelerometer-determined physical activity and self-reported health in a population of older adults (65–85 years): A cross-sectional study. BMC Public Health.

[90] Berkemeyer K., Wijndaele K., White T., Cooper A.J.M., Luben R., Westgate K., Griffin S.J., Khaw K.T., Wareham N.J., Brage S. (2016). The descriptive epidemiology of accelerometer-measured physical activity in older adults. Int. J. Behav. Nutr. Phys. Act..

[91] Jeffries B.J., Sartini C., Shiroma E., Whincup P.H., Wannamethee S.G., Lee I.M. (2015). Duration and breaks in sedentary behavior: Accelerometer data from 1566 community-dwelling older men (British Regional Heart Study). Br. J. Sports Med..

[92] Arnardottir N.Y., Koster A., Van Domelen D.R., Brychta R.J., Caserotti P., Eiriksdottir G., Sverrisdottir J.E., Launer L.J., Gudnason V., Johannsson E. (2013). Objective measurements of daily physical activity patterns and sedentary behavior in older adults: Age, gene/environment susceptibility-Reykjavik study. Age Ageing.

[93] Gao K.L., Tsang W.W.N. (2008). Use of accelerometry to quantify the physical activity level of the elderly. Hong Kong Physiother. J..

[94] Chen T., Narazaki K., Honda T., Chen S., Haeuchi Y., Nofuji Y.Y., Matsuo E., Kumagai S. (2015). Tri-axial accelerometer-determined daily physical activity and sedentary behavior of suburban community-dwelling older Japanese adults. J. Sports Sci. Med..

[95] Zhu W., Chi A., Sun Y. (2016). Physical activity among older Chinese adults living in urban and rural areas: A review. J. Sports Health Sci..

[96] Evenson K.R., Morland K.B., Wen F., Scanlin K. (2014). Physical activity and sedentary behavior among adults 60 years and older: New York residents compared to a national sample. J. Aging Phys. Act..

[97] Gorman E., Hanson H.M., Yang P.H., Khan K.M., Liu-Ambrose T., Ashe M.C. (2014). Accelerometry analysis of physical activity and sedentary behavior in older adults: A systematic review and data analysis. Eur. Rev. Aging Phys. Act..

[98] Barnett A., van den Hoek D., Barnett D., Cerin E. (2016). Measuring moderate-intensity walking in older adults using the ActiGraph Accelerometer. BMC Geriatr..

[99] Whitcher L., Papadopoulos C. (2014). Accelerometer derived activity counts and oxygen consumption between young and older Individuals. J. Aging Res..

[100] Bourke A.K., Masse F., Arami A., Aminian K., Healy M., Nelson J. Energy expenditure estimation using accelerometry and heart rate for multiple sclerosis and healthy older adults: Validation against a mobile metabolic energy expenditure measurement system. Proceedings of the International Conference on Wearable and Implantable Body Sensor Networks Workshops.

[101] Brandon L.J., Ross D.A., Sanford J.A., Lloyd A. (2004). Predicting oxygen uptake in older adults using lower-limb accelerometer measures. J. Rehabil. Res. Dev..

[102] Najafi B., Aminian K., Paraschiv-Ionescu A., Loew F., Bula C.J., Robert P. (2003). Ambulatory system for human motion analysis using a kinematic sensor: Monitoring of daily physical activity in the elderly. IEEE Trans. Biomed. Eng..

[103] Culhane K.M., Lyons G.M., Hilton D., Grace P.A., Lyons D. (2004). Long-term mobility monitoring of older adults using accelerometers in a clinical environment. Clin. Rehabil..

[104] Paiyarom S., Tungamchit P., Keinprasit R., Kayasith P. Activity monitoring system using dynamic time warping for the elderly and disabled people. Proceedings of the International Conference on Computer, Control and Communication.

[105] Kang D.W., Choi J.S., Lee J.W., Chung S.C., Park S.J., Tack G.R. (2010). Real-time elderly activity monitoring system based on a tri-axial accelerometer. Disabil. Rehabil. Assist. Technol..

[106] Khan A.M., Lee Y.K., Lee S., Kim T.S. (2010). Accelerometer’s position independent physical activity recognition system for long-term activity monitoring in the elderly. Med. Biol. Eng. Comput..

[107] Sekine M., Tamura T., Fujimoto T., Fukui T. Classification of walking pattern using acceleration waveform in-elderly people. Proceedings of the Annual EMBS International Conference on Engineering in Medicine and Biology Society.

[108] Muscillo R., Schmid M., Conforto S., D’Alessio T. (2010). An adaptive Kalman-based Bayes estimation technique to classify locomotor activities in young and elderly adults through accelerometers. Med. Eng. Phys..

[109] Weiss A., Brozgol M., Giladi N., Hausdorff J.M. (2016). Can a single lower trunk body-fixed sensor differentiate between level-walking and stair descent and ascent in older adults? Preliminary findings. Med. Eng. Phys..

[110] Chernbumroong S., Cang S., Yu H. (2015). Genetic algorithm-based classifiers fusion for multisensor activity recognition of elderly people. IEEE J. Biomed. Health Inform..

[111] Ul Alam M.A., Roy N., Holmes S., Gangopadhyay A., Galik E. Automated functional and behavioural health assessment of older adults with dementia. Proceedings of the IEEE Conference on Connected Health: Applications, Systems and Engineering Technologies (CHASE).

[112] Sasaki J.E., Hickey A.M., Staudenmayer J.W., John D., Kent J.A., Freedson P.S. (2016). Performance of activity classification algorithms in free-living older adults. Med. Sci. Sports Exerc..

[113] Papadopoulos A., Vivaldi N., Crump C., Silvers C.T. (2015). Differentiating walking from other activities of daily living in older adults using wrist-based accelerometers. Curr. Aging Sci..

[114] Ganea R., Paraschiv-Ionescu A., Salarian A., Büla C., Martin E., Rochat S., Hoskovec C., Piot-Ziegler C., Aminian K. Kinematics and dynamic complexity of postural transitions in frail elderly subjects. Proceedings of the Annual International Conference on IEEE EMBS.

[115] Najafi B., Armstrong D.G., Mohler J. (2013). Novel wearable technology for assessing spontaneous daily physical activity and risk of falling in older adults with diabetes. J. Diabetes Sci. Technol..

[116] Zhang W., Regterschot G.R., Schaabova H., Baldus H., Zijlstra W. (2014). Test-retest reliability of a pendant-worn sensor device in measuring chair rise performance in older persons. Sensors.

[117] Van Lummel R.C., Ainsworth E., Lindemann U., Zijlstra W., Chiari L., Van Campen P., Hausdorff J.M. (2013). Automated approach for quantifying the repeated sit-to-stand using one body fixed sensor in young and older adults. Gait Posture.

[118] Walgaard S., Faber G.S., Van Lummel R.C., Van Dieën J.H., Kingma I. (2016). The validity of assessing temporal events, sub-phases and trunk kinematics of the sit-to-walk movement in older adults using a single inertial sensor. J. Biomech..

[119] Millor N., Lecumberri P., Gomez M., Martinez-Ramirez A., Rodrıguez-Manas L., Garcıa-Garcıa F.J., Izquierdo M. (2013). Automatic evaluation of the 30-s chair stand test using inertial/magnetic-based technology in an older prefrail population. IEEE J. Biomed. Health Inform..

[120] Mariani B., Hoskovec C., Rochat S., Bula C., Penders J., Aminian K. (2010). 3D gait assessment in young and elderly subjects using foot-worn inertial sensors. J. Biomechan..

[121] Rampp A., Barth J., Schulein S., Gaßmann K.G., Klucken J., Eskofier B.M. (2015). Inertial sensor-based stride parameter calculation from gait sequences in geriatric patients. IEEE Trans. Biomed. Eng..

[122] Dadashi F., Mariani B., Rochat S., Büla C.J., Santos-Eggimann B., Aminian K. (2014). Gait and foot clearance parameters obtained using shoe-worn inertial sensors in a large-population sample of older adults. Sensors.

[123] Zhang Y., Beenakker K.G., Butala P.M., Lin C.C., Little T.D., Maier A.B., Stijntjes M., Vartanian R., Wagenaar R.C. Monitoring walking and cycling of middle-aged to older community dwellers using wireless wearable accelerometers. Proceedings of the Annual International Conference on IEEE Engineering in Medicine and Biology Society EMBS.

[124] Atallah L., Wiik A., Lo B., Cobb J.P., Amis A.A., Yang G.Z. (2014). Gait asymmetry detection in older adults using a light ear-worn sensor. Physiol. Meas..

[125] Takenoshita K., Shiozawa N., Onishi J., Makikawa M. Development of a portable acceleration monitor device and its clinical application for the quantitative gait assessment of the elderly. Proceedings of the IEEE 27th Annual International Conference of the Engineering in Medicine and Biology Society.

[126] Chan H., Yang M., Zheng H., Wang H., Sterritt R., McClean S., Mayagoitia R.E. Machine learning and statistical approaches to assessing gait patterns of younger and older healthy adults climbing stairs. Proceedings of the International Conference on Natural Computation (ICNC).

[127] Clermont C.A., Barden J.M. (2016). Accelerometer-based determination of gait variability in older adults with knee osteoarthritis. Gait Posture.

[128] Del Din S., Godfrey A., Rochester L. (2016). Validation of an accelerometer to quantify a comprehensive battery of gait characteristics in healthy older adults and Parkinson’s disease: Toward clinical and at home use. IEEE J. Biomed. Health Inform..

[129] Hartmann A., Luzi S., Murer K., de Bie R.A., de Bruin E.D. (2009). Concurrent validity of a trunk tri-axial accelerometer system for gait analysis in older adults. Gait Posture.

[130] Hartmann A., Murer K., de Bie R.A., de Bruin E.D. (2009). Reproducibility of spatio-temporal gait parameters under different conditions in older adults using a trunk tri-axial accelerometer system. Gait Posture.

[131] Grimpampi E., Oesen S., Halper B., Hofmann M., Wessner B., Mazzà C. (2015). Reliability of gait variability assessment in older individuals during a six-minute walk test. J. Biomech..

[132] Donath L., Faude O., Lichtenstein E., Pagenstert G., Nüesch C., Mündermann A. (2016). Mobile inertial sensor based gait analysis: Validity and reliability of spatiotemporal gait characteristics in healthy seniors. Gait Posture.

[133] Hasomed RehaGait. https://www.hasomed.de/en/rehagait/rehagait/product-overview.html.

[134] Brodie M.A., Coppens M.J., Lord S.R., Lovell N.H., Gschwind Y.J., Redmond S.J., Del Rosario M.B., Wang K., Sturnieks D.L., Persiani M. (2016). Wearable pendant device monitoring using new wavelet‑based methods shows daily life and laboratory gaits are different. Med. Biol. Eng. Comput..

[135] O’Sullivan M., Blake C., Cunningham C., Boyle G., Finucane C. (2009). Correlation of accelerometry with clinical balance tests in older fallers and non-fallers. Age Ageing.

[136] Martinez-Mendez R., Sekine M., Tamura T. (2012). Postural sway parameters using a triaxial accelerometer: Comparing elderly and young healthy adults. Comput. Methods Biomech. Biomed. Eng..

[137] SankarPandi S.K., Dlay S., Woo W.L., Catt M. Predicting disability levels of community dwelling older individuals using single wrist mounted accelerometer. Proceedings of the IEEE-EMBS International Conference on Bioned Health Inform (BHI).

[138] Smith E., Walsh L., Doyle J., Greene B., Blake C. (2016). The reliability of the quantitative timed up and go test (QTUG) measured over five consecutive days under single and dual-task conditions in community dwelling older adults. Gait Posture.

[139] Sheehan K.J., Greene B.R., Cunningham C., Crosby L., Kenny R.A. (2014). Early identification of declining balance in higher functioning older adults, an inertial sensor based method. Gait Posture.

[140] Tung J.Y., Ng H., Moore C., Giangregorio L. (2014). Can we use accelerometry to monitor balance exercise performance in older adults?. Gait Posture.

[141] Chaudhuri S., Thompson H., Demiris G. (2014). Fall detection devices and their use with older adults: A systematic review. J. Geriatr. Phys. Ther..

[142] Schwickert L., Becker C., Lindemann U., Maréchal C., Bourke A., Chiari L., Helbostad J.L., Zijlstra W., Aminian K., Todd C. (2013). FARSEEING Consortium and the FARSEEING Meta Database Consensus Group, Fall detection with body-worn sensors: A systematic review. Z. Gerontol. Geriatr..

[143] Klenk J., Becker C., Lieken F., Nicolai S., Maetzler W., Alt W., Zijlstra W., Hausdorff J.M., van Lummel R.C., Chiari L. (2011). Comparison of acceleration signals of simulated and real-world backward falls. Med. Eng. Phys..

[144] Kangas M., Vikman I., Nyberg L., Korpelainen R., Lindblom J., Jamsa T. (2012). Comparison of real-life accidental falls in older people with experimental falls in middle-aged test subjects. Gait Posture.

[145] Bloch F., Gautier V., Noury N., Lundy J.E., Poujaud J., Claessens Y.E., Rigaud A.S. (2011). Evaluation under real-life conditions of a stand-alone fall detector for the elderly subjects. Ann. Phys. Rehabil. Med..

[146] Tamrat T., Griffin M., Rupcic S., Kachnowski S., Taylor T., Barfield J. Operationalizing a wireless wearable fall detection sensor for older adults. Proceedings of the International Conference on Pervasive Computing Technologies for Healthcare (Pervasive Health) and Workshops.

[147] Palmerini L., Bagalà F., Zanetti A., Klenk J., Becker C., Cappello A. (2015). A wavelet-based approach to fall detection. Sensors.

[148] Bourke A.K., Klenk J., Schwickert L., Aminian K., Ihlen E.A.F., Mellone S., Helbostad H.J., Chiari L., Becker C. Fall detection algorithms for real-world falls harvested from lumbar sensors in the elderly population: A machine learning approach. Proceedings of the IEEE International Conference on Engineering in Medicine and Biology Society.

[149] Liu J., Kanno T., Akashi M., Chen W., Wei D., Wu G., Takeda N. Patterns of bipedal walking on tri-axial acceleration signals and their use in identifying falling risk of older people. Proceedings of the IEEE International Conference on Computer and Information Technology (CIT).

[150] Marschollek M., Wolf K.H., Gietzelt M., Nemitz G., Meyer zu Schwabedissen H., Haux R. Assessing elderly persons’ fall risk using spectral analysis on accelerometric data – a clinical evaluation study. Proceedings of the 30th Annual International Conference of the IEEE Engineering in Medicine and Biology Society.

[151] Barrett A., O’Connor M., Culhane K., Finucane A.M., Mulkerrin E., Lyons D., O’Laighin G. Accelerometer versus footswitch evaluation of gait unsteadiness and temporal characteristics of gait in two elderly patient groups. Proceedings of the 30th Annual International Conference of the IEEE Engineering in Medicine and Biology Society.

[152] Jiang S., Zhang B., Wei D. The elderly fall risk assessment and prediction based on gait analysis. Proceedings of the 2011 IEEE 11th International Conference on Computer and Information Technology (CIT).

[153] Doheny E.P., Fan C.W., Foran T., Greene B.R., Cunningham C., Kenny R.A. An instrumented sit-to-stand test used to examine differences between older fallers and non-fallers. Proceedings of the 2011 Annual International Conference of the IEEE Engineering in Medicine and Biology Society.

[154] Doheny E.P., McGrath D., Greene B.R., Walsh L., McKeown D., Cunningham C., Crosby L., Kenny R.A., Caulfield B. Displacement of centre of mass during quiet standing assessed using accelerometry in older fallers and non-fallers. Proceedings of the 2012 Annual International Conference of the IEEE Engineering in Medicine and Biology Society.

[155] Ishigaki N., Kimura T., Usui Y., Aoki K., Narita N., Shimizu M., Hara K., Ogihara N., Nakamura K., Kato H. (2011). Analysis of pelvic movement in the elderly during walking using a posture monitoring system equipped with a triaxial accelerometer and a gyroscope. J. Biomech..

[156] Paterson K., Hill K., Lythgo N. (2011). Stride dynamics, gait variability and prospective falls risk in active community dwelling older women. Gait Posture.

[157] Riva F., Toebes M.P.J., Pijnappels M., Stagni R., van Dieen J.H. (2013). Estimating fall risk with inertial sensors using gait stability measures that do not require step detection. Gait Posture.

[158] Brodie M.A., Menz H.B., Smith S.T., Delbaere K., Lord S.R. (2015). Good lateral harmonic stability combined with adequate gait speed is required for low fall risk in older people. Gerontology.

[159] Ejupi A., Brodie M., Lord S.R., Annegarn J., Redmond S.J., Delbaere K. (2016). Wavelet-based sit-to-stand detection and assessment of fall risk in older people using a wearable pendant device. IEEE Trans. Biomed. Eng..

[160] Ihlen E.A., Weiss A., Bourke A., Helbostad J.L., Hausdorff J.M. (2016). The complexity of daily life walking in older adult community-dwelling fallers and non-fallers. J. Biomech..

[161] Brodie M.A., Lord S.R., Coppens M.J., Annegarn J., Delbaere K. (2015). Eight-week remote monitoring using a freely worn device reveals unstable gait patterns in older fallers. IEEE Trans. Biomed. Eng..

[162] Van Schooten K.S., Pijnappels M., Rispens S.M., Elders P.J.M., Lips P., Daffertshofer A., Beek P.J., van Dieën J.H. (2016). Daily-life gait quality as predictor of falls in older people: A 1-year prospective cohort study. PLoS ONE.

[163] Howcroft J., Kofman J., Lemaire E.D., McIlroy W.E. (2016). Analysis of dual-task elderly gait in fallers and non-fallers using wearable sensors. J. Biomech..

[164] Hynes M., Wang H., Kilmartin L., McCarrick E. Monitoring of activity level s of the elderly in home and community environments using off the shelf cellular handsets. Proceedings of the 2010 Digest of Technical Papers International Conference on Consumer Electronics (ICCE).

[165] Hewson D.J., Jaber R., Chkeir A., Hammoud A., Gupta D., Bassement J., Vermeulen J., Yadav S., de Witte L., Duchêne J. Development of a monitoring system for physical frailty in independent elderly. Proceedings of the 2013 35th Annual International Conference of the IEEE Engineering in Medicine and Biology Society.

[166] Tang Y., Wang S., Chen Y., Chen Z. PPCare: A personal and pervasive health care system for the elderly. Proceedings of the 2012 9th International Conference on Ubiquitous Intelligence & Computing and 9th International Conference on Autonomic & Trusted Computing (UIC/ATC).

[167] Noury N., Quach K.A., Berenguer M., Bouzi M.J., Teyssier H. A feasibility study of using a smartphone to monitor mobility in elderly. Proceedings of the 2012 IEEE 14th International Conference on e-Health Networking, Applications and Services (Healthcom).

[168] Capela N.A., Lemaire E.D., Baddour N. (2015). Feature selection for wearable smartphone-based human activity recognition with able bodied, elderly, and stroke patients. PLoS ONE.

[169] Cerrito A., Bichsel L., Radlinger L., Schmid S. (2015). Reliability and validity of a smartphone-based application for the quantification of the sit-to-stand movement in healthy seniors. Gait Posture.

[170] Galán-Mercant A., Cuesta-Vargas A.I. (2013). Differences in trunk accelerometry between frail and nonfrail elderly persons in sit-to-stand and stand-to-sit transitions based on a mobile inertial sensor. JMIR Mhealth Uhealth.

[171] Fontecha J., Navarro J., Hervas R., Bravo J. (2013). Elderly frailty detection by using accelerometer-enabled smartphones and clinical information records. Pers. Ubiquit. Comput..

[172] Vathsangam H., Sukhatme G.S. Using phone-based activity monitors to promote physical activity in older adults: A pilot study. Proceedings of the IEEE Healthcare Innovation Conference(HIC).

[173] Cadmus-Bertram L.A., Marcus B.H., Patterson R.E., Parker B.A., Morey B.L. (2015). Randomized trial of a Fitbit-based physical activity intervention for women. Am. J. Prev. Med..

[174] Nicklas B.J., Gaukstern J.E., Beavers K.M., Newman J.C., Leng X., Rejeski W.J. (2014). Self-monitoring of spontaneous physical activity and sedentary behavior to prevent weight regain in older adults. Obesity.

[175] Sookhai L., Coppola J.F., Gaur C. Intergenerational activity tracker program: Impact with health related outcomes on older adults. Proceedings of the IEEE Long Island Systems, Applications and Technology Conference (LISAT).

[176] Holzinger A., Searle G., Pruckner S., Steinbach-Nordmann S., Kleinberger T., Hirt E., Temnitzer J. Perceived usefulness among elderly people: Experiences and lessons learned during the evaluation of a wrist device. Proceedings of the IEEE International Conference on Pervasive Computing Technologies for Healthcare (PervasiveHealth).

[177] Rasche P., Wille M., Theis S., Schafer K., Schlink C.M., Mertens A. Activity tracker and elderly. Usability and motivation of mobile healthcare in the context of elderly people. Proceedings of the IEEE International Conference on Computer and Information Technology; Ubiquitous Computing and Communications; Dependable, Autonomic and Secure Computing; Pervasive Intelligence and Computing (CIT/IUCC/DASC/PICOM).

[178] Schlomann A., von Storch K., Rasche P., Rietz C. (2016). Means of motivation or of stress? The use of fitness trackers for self-monitoring by older adults. HBScience.

[179] Preusse K.C., Mitzner T.L., Fausset C.B., Rogers W.A. Activity monitoring technologies and older adult users: Heuristic analysis and usability assessment. Proceedings of the International Symposium Human Factors and Ergonomics in Health Care.

[180] Preusse K.C., Mitzner T.L., Fausset C.B., Rogers W.A. (2017). Older adults’ acceptance of activity trackers. J. Appl. Gerontol..

[181] O’Brien T., Troutman-Jordan M., Hathaway D., Armstrong S., Moore M. (2015). Acceptability of wristband activity trackers among community dwelling older adults. Geriatr. Nurs..

[182] Mercer K., Giangregorio L., Schneider E., Chilana P., Li M., Grindrod K. (2016). Acceptance of commercially available wearable activity trackers among adults aged over 50 and with chronic illness: A mixed-methods evaluation. JMIR Mhealth Uhealth.

[183] Tocci F.L., Morey M.C., Caves K.M., Deberry J., Leahy G.D., Hall K. (2016). Are older adults ready for wireless physical activity tracking devices? A comparison of commonly used tracking devices. J. Am. Geriatr. Soc..

[184] McMahon S.K., Lewis B., Oakes M., Guan W., Wyman J.F., Rothman A.J. (2016). Older adults’ experiences using a commercially available monitor to self-track their physical activity. JMIR Mhealth Uhealth.

[185] Wang J., Carroll D., Peck M., Myneni S., Gong Y. (2016). Mobile and wearable technology needs for aging in place: Perspectives from older adults and their caregivers and providers. Stud. Health Technol. Inform..

[186] Fausset C.B., Mitzner T.L., Price C.E., Jones B.D., Fain W.B., Rogers W.A. Older adults’ use of and attitudes toward activity monitoring technologies. Proceedings of the Human Factors and Ergonomics Society Annual Meeting.

[187] Lebron J., Escalante K., Coppola J., Gaur C. Activity tracker technologies for older adults: Successful adoption via intergenerational telehealth. Proceedings of the IEEE Long Island Systems, Applications and Technology Conference (LISAT).

[188] Buchem I., Merceron A., Kreutel J., Haesner M., Steinert A. Gamification designs in wearable enhanced learning for healthy ageing. Proceedings of the IEEE International Conference on Interactive Mobile Communication Technologies and Learning (IMCL).

[189] Batsis J.A., Naslund J.A., Gill L.E., Masutani R.K., Agarwal N., Bartels S.J. (2016). Use of a wearable activity device in rural older obese adults: A pilot study. Gerontol. Geriatr. Med..

[190] Attick J., Berekdar D., Conklin R., Villalon F., Coppola J.F., Gaur C. (2016). Wearable technology and older adult motivation: Urban versus suburban communities. Proceedings of Student-Faculty Research Day, 6 May 2016.

[191] Paul S.S., Tiedemann A., Hassett L.M., Ramsay E., Kirkham C., Chagpar S., Sherrington C. (2015). Validity of the Fitbit activity tracker for measuring steps in community-dwelling older adults. BMJ Open Sport Exerc. Med..

[192] Simpson L.A., Eng J.J., Klassen T.D., Lim S.B., Louie D.R., Parappilly B., Sakakibara B.M., Zbogar D. (2015). Capturing step counts at slow walking speeds in older adults: Comparison of ankle and waist placement of measuring device. J. Rehabil. Med..

[193] Ehrler F., Weber C., Lovis C. (2016). Positioning commercial pedometers to measure activity of older adults with slow gait: At the wrist or at the waist?. Transforming Healthcare with the Internet of Things.

[194] Stahl S.T., Insana S.P. (2014). Caloric expenditure assessment among older adults: Criterion validity of a novel accelerometry device. J. Health Psychol..

[195] Floegel T.A., Florez-Pregonero A., Hekler E.B., Buman M.P. (2017). Validation of consumer-based hip and wrist activity monitors in older adults with varied ambulatory abilities. J. Gerontol. A Biol. Sci. Med. Sci..

[196] Taraldsen K., Askim T., Sletvold O., Einarsen E.K., Bjastad K.G., Indredavik B., Helbostad J.L. (2011). Evaluation of a body-worn sensor system to measure physical activity in older people with impaired function. Phys. Ther..

[197] Lauritzen J., Munoz A., Luis Sevillano J., Civit A. (2013). The usefulness of activity trackers in elderly with reduced mobility: A case study. Stud. Health Technol. Inform..

[198] AARP—Project Catalyst, Building a Better Tracker: Older Consumers Weigh in on Activity and Sleep Monitoring Devices. http://www.aarp.org/content/dam/aarp/home-and-family/personal-technology/2015-07/innovation-50-project-catalyst-tracker-study-AARP.pdf.

[199] Lively. http://www.mylively.com/.

[200] GrandCare. https://www.grandcare.com/.

[201] Healthsense. http://healthsense.com/.

[202] CareInnovations. http://www.careinnovations.com/quietcare/.

[203] Evermind. https://evermind.us/evermind-for-family-caregivers.

[204] Peakfoqus. http://peakfoqus.spacecrafted.com/.

[205] Mindme. http://www.mindme.care/mindme-locate.html.

[206] SmartSole. http://gpssmartsole.com/gpssmartsole/.

[207] iTraq. https://www.itraq.com/.

[208] Proximity. http://www.proximitycare.co.uk/.

[209] Pocketfinder. http://pocketfinder.com/.

[210] Comfort Zone Check-In Mobile. https://itunes.apple.com/us/app/comfort-zone-check-in-mobile/id565267103?mt=8.

[211] V.ALRT. http://vsnmobil.com/products/v-alrt/.

[212] Tweri. http://www.tweri.com/home.aspx.

[213] Lok8u. http://www.lok8u.com/freedom/.

[214] PAL. http://www.projectlifesaver.org/Pal-info/.

[215] SafeLink GPS. http://wp.safelinkgps.com/#aboutus.

[216] RevolutionaryTracker. http://www.revolutionarytracker.com/.

[217] Bluewater Security Professionals, LLC. http://www.bluewatersecurityprofessionals.com/elderlytracking.htm.

[218] Everon. http://www.everon.fi/en/solutions/vega-gps-safety-solution-and-bracelet.

[219] Limmex. https://www.limmex.com/intl/en.

[220] mCareWatch. http://mcarewatch.com.au/products/smw-soteria-mobile-watch/.

[221] CleverCare. http://clevercare.co.nz/clevercare-as-a-medical-alarm/.

[222] CarePredict. https://www.carepredict.com/.

[223] UnaliWear. http://www.unaliwear.com/.

[224] Allen Band. http://theallenband.com/.

[225] IN LIFE. http://inlife-projekt.si/.

[226] WatchRx. http://www.watchrx.io/.

[227] EasyM2M. http://www.easym2m.in/.

[228] Reemo. http://getreemo.com/for-seniors-families/.

[229] Omate S3. https://www.omate.com/s3/.

[230] Toshiba to release wristband-like biological sensor. https://www.japantoday.com/category/technology/view/toshiba-to-release-wristband-like-biological-sensor-with-many-functions.

[231] Haier. http://haiersmartwatch.com/.

[232] Secom readies elderly monitoring service using smart wristband. http://asia.nikkei.com/Tech-Science/Tech/Secom-readies-elderly-monitoring-service-using-smart-wristband.

[233] Amulyte. http://www.amulyte.com/.

[234] AdhereTech. https://adheretech.com/.

[235] Preventice Services. http://www.preventicesolutions.com/services/body-guardian-heart.html.

[236] Sense4Care. http://www.sense4care.com/en.

[237] ActiveProtective. http://activeprotective.com/.

[238] StepsCount. https://www.stepscount.com/.

[239] FSA Store. https://fsastore.com/FSA-Eligibility-List/F/Fitness-Tracker-E792.aspx.

[240] FDA. http://www.fda.gov/downloads/medicaldevices/deviceregulationandguidance/guidancedocuments/ucm429674.pdf.

[241] iRythm. http://www.irhythmtech.com/.

[242] BioSensics. http://www.biosensics.com/.

[243] MobiHealth New. http://www.mobihealthnews.com/36158/va-to-reimburse-for-certain-clinical-activity-trackers.

[244] PRNewswire Timetric, Insight Report: Digital Innovation in Insurance. http://www.prnewswire.com/news-releases/insight-report-digital-innovation-in-insurance-300160076.html.

[245] Strategy Meets Action. https://strategymeetsaction.com/news-and-events/sma-blog/wearables-in-insurance-balancing-value-and-vulnerabilities/.

[246] PwC The Wearable Future. https://www.pwc.com/us/en/technology/publications/assets/pwc-wearable-tech-design-oct-8th.pdf.

[247] Accenture Technology Vision for Insurance 2015. https://www.accenture.com/ie-en/insight-technology-vision-insurance-2015.aspx.

[248] Life Happens. https://www.lifehappens.org/industry-resources/agent/barometer2016/.

[249] EY 2016 Sensor Data Survey. http://www.ey.com/gl/en/industries/financial-services/insurance/ey-2016-sensor-data-survey.

[250] Abraham M. Wearable technology: A health-and-care actuary’s perspective.

[251] EY PAYL—Wearable Trends. http://www.ey.com/Publication/vwLUAssets/EY-wearable-trends/$FILE/EY-wearable-trends.pdf.

[252] Capgemini Wearable Devices and Their Applicability in the Life Insurance Industry. https://www.capgemini.com/resource-file-access/resource/pdf/wearable_devices_and_their_applicability_in_the_life_insurance_industry.pdf.

[253] RolandBerger Internet of Things and Insurance. http://www.the-digital-insurer.com/wp-content/uploads/2015/06/537-Roland_Berger_Internet_of_Things_and_insurance_20150513.pdf.

[254] ABIresearch. https://www.abiresearch.com/press/corporate-wellness-is-a-13-million-unit-wearable-w/.

[255] John Hancock. https://www.johnhancockinsurance.com/life/John-Hancock-Vitality-Program.aspx.

[256] Oscar Insurance Corp. https://www.hioscar.com/news/post/119375641948/building-better-rewards-step-by-step.

[257] Vitality. https://www.vitality.co.uk/.

[258] AIA Vitality. https://www.aiavitality.com.au/vmp-au/.

[259] Medibank. https://www.medibank.com.au/livebetter/fitbit/.

[260] AXA France. https://www.axa.fr/mutuelle-sante/partenariat-withings/jeu-pulse.html.

[261] MLC. https://www.mlc.com.au/personal/important-updates/on-track.

[262] Discovery. https://www.discovery.co.za/portal/individual/vitality-fitness-points-sep-2016.

[263] Manulife. http://www.manulife.com/Master-Article-Detail?content_id=a0Q5000000Js8m5EAB.

[264] Highmark Health. https://blog.highmarkhealth.org/member-discounts-walkadoo-smart-step-pebble-fun-way-to-be-more-active/.

[265] Momentum. https://www.momentum.co.za/for/you/multiply/what.

[266] Ping An. http://health.pingan.com/en/chanpinlist/healthProduction.shtml.

[267] Blue Cross Blue Shield. https://www.blue365deals.com/.

[268] Silver & Fit. https://www.silverandfit.com/.

[269] Aetna. http://investor.aetna.com/phoenix.zhtml?c=110617&p=irol-newsArticle&ID=2206242.

[270] Phoenix. https://www.phoenixwm.phl.com/public/index.jsp.

[271] APPIRIO. https://appirio.com/pressroom/news/company-saved-300k-insurance-giving-employees-fitbits.

[272] BP Life Benefits. http://hr.bpglobal.com/LifeBenefits/Sites/core/BP-Life-benefits/Employee-benefits-handbook/BP-Medical-Program/How-the-BP-Medical-Program-works/Health-Savings-OOA-Option-summary-chart/BP-Wellness-Program.aspx.

[273] UnitedHealthcare. https://www.uhc.com/news-room/2016-news-release-archive/wearable-devices-wellness-program.

[274] dorsaVi. http://dorsavi.com/wp-content/sharelink/20151119-allianz-monash-health-and-dorsavi-team-up-4bf5215070561c796111b0119553d84b.pdf.

[275] Humana. https://www.humana.com/employer/products-services/wellness-programs/go365.

[276] Wang Z., Yang Z., Dong T. (2017). A review of wearable technologies for elderly care that can accurately track indoor position, recognize physical activities and monitor vital signs in real time. Sensors.

[277] Helbostad J.L., Vereijken B., Becker C., Todd C., Taraldsen K., Pijnappels M., Aminian K., Mellone S. (2017). Mobile health applications to promote active and healthy ageing. Sensors.

[278] Godfrey A. (2017). Wearables for independent living in older adults: Gait and falls. Maturitas.

